# Consumer acceptance among Dutch and German students of insects in feed and food

**DOI:** 10.1002/fsn3.2006

**Published:** 2020-12-01

**Authors:** Natalia Naranjo‐Guevara, Michelle Fanter, Anna Maria Conconi, Sonja Floto‐Stammen

**Affiliations:** ^1^ Research Group Business Innovation Fontys University of Applied Sciences Venlo The Netherlands

**Keywords:** entomophagy, environmental benefits, health benefits, novel protein sources, visual acceptance, willingness to accept

## Abstract

Due to the environmental and nutritional benefits of insects, their consumption would be one of the solutions to feed the growing human population. Despite the increasing interest in the use of insects as food and feed, consumer acceptance is the major obstacle to successful implementation in Western countries and we studied the factors that influence consumer acceptance in a group of university students from Germany and the Netherlands. In this exploratory research, a survey was conducted (*n* = 222). Socio‐demographic and psychological factors were established from a theoretical review. In addition, we elaborated on questions regarding information on the health and environmental benefits of consuming insects. Initially, the data obtained are presented through descriptive statistics. The influence of the socio‐demographic and psychological factors, and the information on the willingness to accept insects as animal feed and human food was analyzed using correlations and multiple linear regressions. Results showed more willingness to accept insects as animal feed than in human food. The acceptance among German and Dutch students seems to be driven by issues similar to those in other European countries, such as visual aspects and knowledge about the benefits. The effect of the information on willingness constitutes an important finding of this study, especially for the use of insects in animal feed, since most of the previous studies have focused on the use of insects as human food. Our data support the need to inform and educate consumers about the environmental and health benefits of entomophagy. We conclude that effective efforts to implement entomophagy could increase the level of familiarity with the insect food and inform (or educate) consumers about its benefits. Insights from this study are useful to address studies focusing on specific segments of possible early adopters and consequently addressing communication strategies in this market segmentation.

## Graphical abstract

Data about insights into the consumer's acceptance towards insects in feed and food is presented. Rather than invest efforts in reducing neophobia and disgust, it could be more effectively to increase the level of familiarity with the dishes and interest about entomophagy.

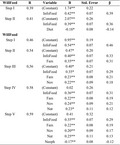

## 1 INTRODUCTION

The growing world population represents a big challenge for the current food supply. An expected growth to 9 billion people by 2050 is creating a necessity for an increase in food production by 70%. However, this demand is placing heavy pressure on limited resources such as land, energy, and water (FAO, 2009; Van Huis et al., 2013). To face these problems, current food production needs to be re‐evaluated, and a sustainable and efficient system introduced (Orsi et al., 2019). The conventional production of animal protein in Western countries requires significant resources (Hartmann et al., 2015). For instance, 70% of all agricultural land worldwide is used for livestock production, mainly for the cultivation of feed (IPIFF, 2019). Moreover, livestock contributes enormously to greenhouse gas emissions (Hedenus et al., 2014). One of the reasons is that the most commonly used protein source to feed livestock is soybean meal (Böhmerle & Pabst, 2018). In the European Union (EU), the dependency on the import of plant‐based protein, such as soybean, for use in animal feed is estimated at around 70% (De Visser et al., 2014; FEFAC, 2019). Thus, adequate animal‐based proteins will become scarcer in the future, and the need to change to a diet less dependent on animal protein is evident.

Insects can provide proteins for human consumption, directly as a food, or indirectly as livestock feed. Consequently, since 2003, the Food and Agriculture Organization of the United Nations, FAO, has promoted insects as an alternative protein source for humans and livestock (Van Huis et al., 2013). Multiple nutritional environmental and economic advantages have been highlighted with respect to insect farming. For example, insects meet animals’ and humans’ dietary requirements due to their nutrient profiles, essential amino acids, fats, vitamins, and essential minerals (Makkara et al., 2014; Rumpold & Schlüter, 2013; Van Huis et al., 2013). In comparison with conventional livestock, insects have a higher feed conversion rate to biomass and a higher fecundity (Rumpold & Schlüter, 2013). Furthermore, they do not require extensive land‐based activities, which results in fewer greenhouse gas emissions (Lammers et al., 2019; Oonincx et al., 2010). Insects are very efficient in converting low‐quality input into a high protein output and stimulate a circular economy (Rumpold & Schlüter, 2013). Positive results have been observed in terms of animal health and performance, gut health, and product quality (reviewed by Sogari, et al., 2019). Additionally, insects may also pose a lower risk of transmitting zoonotic infections to humans, livestock, and wildlife (Dicke et al., 2020; Van Huis et al., 2013). In this context, the consumption of insects (better known as entomophagy) could be one of the ways to enhance food and feed security in a sustainable manner.

Although entomophagy is accepted in several countries worldwide, mainly in South‐East Asia and South America (Van Huis et al., 2013), there is a major attitudinal barrier, especially in Western societies (DeFoliart, 1999; La Barbera et al., 2020). Consumer acceptance has been identified as one of the most important impediments to adopting insects as an alternative resource and the biggest challenge for the insect industry today (Mancini et al., 2019; Van Huis et al., 2013; Verbeke, 2015). Therefore, research is still necessary in this regard. On the one hand, there is a substantial lack of knowledge about the acceptance of insects as animal feed (indirect entomophagy) and the possible factors affecting it (La Barbera et al., 2020; Sogari, et al., 2019). Such acceptance is likely to determine the future success of using insect‐based feed, as well as the foods obtained from animals raised on insect‐based feed (Verbeke, 2015).

On the other hand, studies on consumer acceptance regarding insects as food (direct entomophagy) are better documented (Gmuer et al., 2016; Hartmann & Siegrist, 2016; Tan et al., 2015; Tan et al., 2016; Verbeke, 2015). Such research has shown that most Western people have a negative attitude toward entomophagy, mostly linked to disgust and unfamiliarity (De Boer, Schösler, & Boersema, 2014Cicatiello et al., 2016; de‐Magistris et al., 2015; Lensvelt & Steenbekkers, 2014; Tan, et al., 2016; Van Huis, 2020). Nevertheless, in their work, Sogari, et al. (2019) comment that a change in consumer attitudes toward entomophagy has been observed in Western societies since people have begun to be interested in healthier, more environmentally friendly diets, and more sustainable protein sources. However, the main challenge for the success of the edible insects sector concerns gaining a better comprehension of how to overcome rejection by examining the factors that drive people to accept the eating of insects (directly and indirectly).

In the search for strategies to speed up the process of being ready to accept insects as animal feed and human food, we conducted an exploratory study. The aim was to provide insight into the factors that influence consumer acceptance regarding insects in feed and food. This study was based on existing literature that examines Western consumers' willingness to adopt entomophagy, and we applied it in a German and Dutch context. By means of a survey, we analyze the willingness to accept insects as animal feed and human food as two dependent variables in a sample that consisted of university students.

### 1.1 Theoretical framework

Recently, an increasing amount of scientific literature has mainly focused on the study of Westerners' acceptability (Gmuer et al., 2016; Hartmann & Siegrist, 2016; Tan et al., 2015, 2016; Van Huis, 2020; Verbeke, 2015). However, there is less research concerning the point of view of consumers toward the use of insects as livestock feed (Sogari, et al., 2019) and only few works can be cited such as Verbeke (2015) and Bazoche and Sylvaine (2016). Consumer acceptance has been studied in the light of factors such as socio‐demographics and psychology (food neophobia, visual aspects of the food—familiarity and visibility—health and the environment) as well as the role of information, which will be described below.

#### 1.1.1 Socio‐demographic factors

Socio‐demographic factors such as age, gender, and nationality influence the willingness to eat insects. For example, younger people have been shown to be more willing to eat them than older people (Caparros Megido et al., 2014; Hartmann et al., 2015; Verbeke, 2015). Additionally, it has been shown that men have a more positive attitude toward entomophagy than women (Barsics et al., 2017; Hartmann et al., 2015; Menozzi et al., 2017a, 2017b; Schlup & Brunner, 2018; Schösler et al., 2012; Tan, et al., 2016; Tan, et al., 2016; Van Huis, 2020; Verbeke, 2015). Vartiainen et al. (2020) found that women, students, those under 25 years of age, those living in rural areas, and those who had no earlier experience of eating insects showed less intention to consume insect‐based foods. Generally, it seems that the most probable early adopters are young men with a high educational level (reviewed by Mancini et al., 2019).

The willingness to consume insects is related to culture, and attitudes toward insect food vary across European regions (Deroy et al., 2015; Menozzi et al., 2017a, 2017b; Sogari, et al. (2019); Verneau et al., 2016). For instance, Piha et al. (2018) reported that Finnish and Swedish consumers had a more positive attitude than Germans and Czechs. Menozzi et al. (2017a); Menozzi et al. (2017b) and Sogari, et al. (2019) applied a survey to students from the Southern, Central, and Northern regions of Italy. Their respondents from Southern Italian regions, an area of strong culinary traditions, demonstrated a lower intention to eat products containing insects than those from Central and Northern Italian regions. In their interviews with 231 young adults in Italy and 71 in the Netherlands, Menozzi, Sogari, Veneziani, Simoni, and Mora (2017) showed that the intention to eat a product containing insect flour is significantly higher in the Netherlands.

#### 1.1.2 Psychological factors

Food neophobia, defined as the fear of unfamiliar food (Pliner & Hobden, 1992), has been considered to be an important predictor for understanding consumer acceptance (Hartmann et al., 2015; Hartmann & Siegrist, 2017; La Barbera et al., 2018; Verbeke, 2015). According to the literature, there is a significant negative effect of neophobia on the willingness to eat insects (Hartmann et al., 2015; Hartmann & Siegrist, 2016; Piha et al., 2018; Tan, et al., 2016; Vartiainen et al., 2020; Verbeke, 2015).

One way to reduce the possible impact of food neophobia is to present the insects combined with known ingredients (Caparros Megido et al., 2014; Gmuer et al., 2016; Hartmann et al., 2015; Schösler et al., 2012; Tan et al., 2015; Tan, et al., 2016; Van Thielen et al., 2019). Studies have observed that when insects are incorporated in familiar preparations, they can enhance consumer acceptability of insect‐based products, such as meatballs (Tan et al., 2015), cookies (Hartmann et al., 2015), hamburgers (Caparros Megido et al., 2016; Van Thielen et al., 2019), or pizza made with processed insect protein (Schösler et al., 2012). Therefore, familiarity has been considered a positive predictor of the willingness to eat insects (Hartmann & Siegrist, 2016; Verbeke, 2015).

Likewise, consumers have shown to be more willing to eat products containing less visible or more processed insect ingredients (Caparros Megido et al., 2016; Gmuer et al., 2016; Jensen & Lieberoth, 2019; Menozzi et al., 2017a, 2017b; Schösler et al., 2012; Sogari et al., 2017, 2018). Previous studies suggested integrating “invisible insects” into food preparation and/or to associate them with attractive flavors (Caparros Megido et al., 2016; Lombardi et al., 2019). However, occasionally combining insects with a familiar or more processed product is not enough to enhance the acceptance (Tan, et al., 2016).

Most studies that consider environmental awareness have highlighted that the potential of the insects as a sustainable food could lead to a positive effect on the acceptance of entomophagy (Hartmann & Siegrist, 2018; Menozzi et al., 2017a, 2017b; Tan et al., 2015). Kostecka et al. (2017) claimed that the consumers’ environmental awareness was an important factor for improving the likelihood to eat insects. However, other studies reported that high environmental consciousness does not demonstrate a significantly increased likelihood to consume whole insects (Hartmann et al., 2015; Laureati et al., 2016; Orsi et al., 2019).

#### 1.1.3 Information role

Previous studies have shown that distributing information about the benefits of entomophagy affects consumers' perceptions (Caparros Megido et al., 2016; Hartmann & Siegrist, 2016; Tan, et al., 2016; Verneau et al., 2016) and may enhance their willingness to eat insects (Barsics et al., 2017; Lombardi et al., 2019; Sogari, et al., 2019; Verneau et al., 2016; Verneau et al., 2016). According to Hartmann and Siegrist (2018), information on the environmental impact and the sustainability of insect production could affect consumer acceptance positively. On the other hand, Hartmann et al. (2015) found that German consumers were not motivated by health benefits or sustainability attributes of insect food.

#### 1.1.4 Theoretical model

**FIGURE 1:**
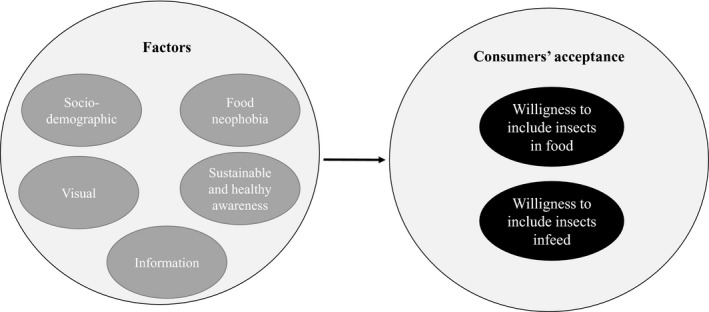
Theoretical model with factors influencing consumer acceptance

In an attempt to determine the factors that influence consumer acceptance regarding insects in feed and food, in a group of young university students from Germany and the Netherlands, a theoretical model is proposed. This was made by combining the most essential factors obtained from previous studies conducted in Europe (Figure 1).

## 2 MATERIAL AND METHODS

### 2.1 Survey

Based on the theoretical framework, a survey was conducted in which each statement was linked to one of the previously described factors that are considered to influence the consumer acceptance of insects in human food or animal feed. Initially, the respondents answered four statements concerning socio‐demographic information. Then, they responded to two statements concerning the willingness to incorporate insects in human food and animal feed, respectively. That was followed by six statements regarding visual acceptability. Thereafter, the respondents answered one statement concerning food neophobia. This was followed by three items about consumers’ environmental and health awareness. Finally, subjects were given information about the nutritional and environmental benefits of using insects as food and feed. Then, they responded to two statements regarding the willingness to eat products from animals fed with insects and two statements regarding the willingness to eat products made from insects. A 4‐point Likert scale was employed in which low values indicated a low level of agreement with the given statement and high values indicated a high level of agreement (Appendix S1). Responses were coded from 1 to 4. The statements did not have a *nonresponse* option, however in some cases (from 0.5% to 3% in different statements) the students were not able to respond. We found this event represents the uncertainty of the sample. Such responses were coded 0.

### 2.2 Socio‐demographic information

The survey took place in Venlo, the Netherlands, a city that is located close to the border with Germany. Two hundred and twenty‐two students (mean age 21 ± 1.3) at Fontys International Business School participated. The sample was represented by 55% and 45% of German and Dutch students, respectively. About 30% of the participants were female, 67% male and 3% belonged to diverse groups or did not respond. Approximately 77% of the participants follow a varied diet with meat and/or fish (VMF), 12% are flexitarian, 6% vegetarian, <1% vegan, and the remaining 4% were uncertain about their diets (Table 1).

**Table 1:** Socio‐demographic information

	Sample population	
	N	%
Nationality		
Germany	122	55.0
The Netherlands	100	45.0
Age		
<18	8	3.6
18–23	199	89.6
24–26	9	4.1
>26	6	2.7
Gender		
No answer	2	0.9
Female	67	30.2
Male	150	67.6
Diverse	3	1.4
Diet		
Varied diet with meat and/or fish	172	77.5
Flexitarian	26	11.7
Vegetarian	14	6.3
Vegan	2	0.9
Uncertain	8	3.6
Total	222	100

### 2.3 Willingness to accept insects as animal feed and human food

Two statements regarding willingness to include insects as animal feed and human food were made. The first concerned animal feed: “Would you agree to eating meat or eggs if the animals were raised with feed containing insect protein?” and the second concerned human food: “Would you agree to eating products containing insects, for example, pancakes or pasta with insect powder?” Using a 4‐point Likert scale (1 = “strongly disagree,” 2 = “disagree,” 3 = “agree,” and 4 = “strongly agree”), respondents indicated their degree of agreement. According to Laureati et al. (2016), the respondents were categorized into three groups in order to gain a better visualization of the data: (i) uncertain (students who did not give an answer); (ii) unwilling (students who answered “strongly disagree” and “disagree”); and (iii) willing (students who answered “agree” and “strongly agree”).

### 2.4 Visual acceptability

**FIGURE 2:**
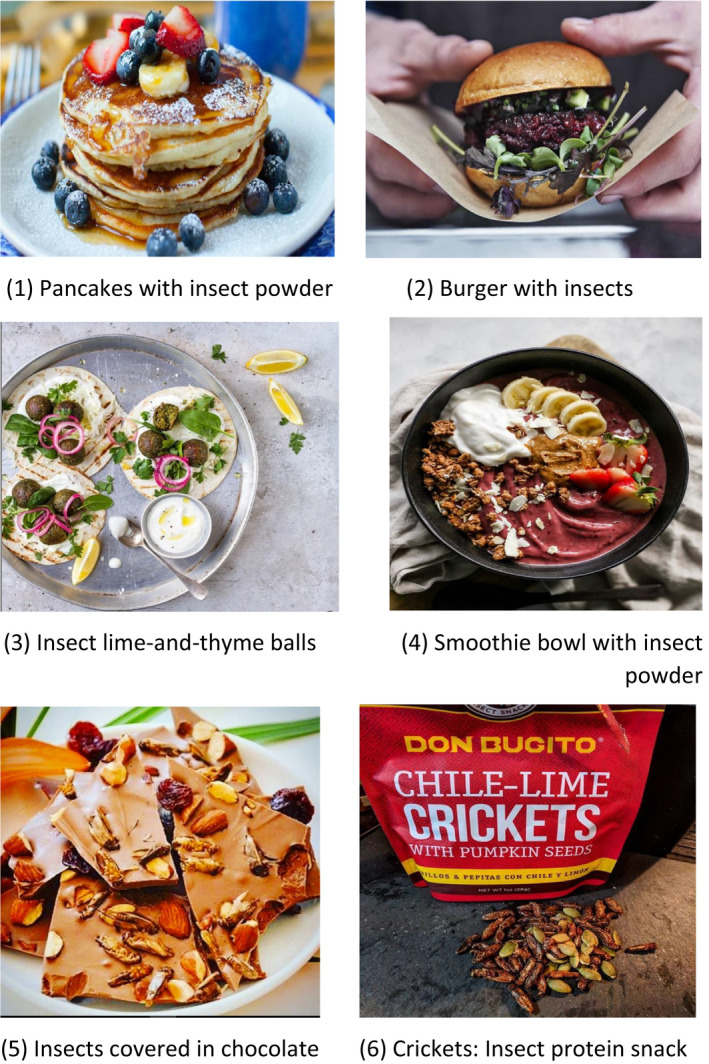
Images of the insect‐based foods shown to the participants in the test of visual acceptability

To assess the students’ acceptance of different insect‐based food, one visual test was performed. Six images of insect‐based dishes are shown in Figure 2. They were classified into three categories: (a) familiarity, which consisted of familiar dishes made with insect ingredients (pancakes with insect powder and burgers); (b) novelty, which consisted of new dishes made with insect ingredients (insect lime‐and‐thyme balls and smoothie bowl with insect powder); and (c) visibility, foods in which insects are visible (insects covered in chocolate and cricket protein snack). The participants indicated the likeliness that they would try the product using a 4‐point Likert scale (1 = “highly unlikely,” 2 = “unlikely,” 3 = “likely,” and 4 = “highly likely”). In the case of unwillingness to try, participants could choose between six options (lack of knowledge; religion; disgust; food safety; none of the above; or other).

### 2.5 Food neophobia

Food neophobia was assessed based on a statement in which respondents chose the one that best suited them from among three options: “I often try new foods and am curious about new products”; “I am open to new foods, but it should be close to what I already know”; and “I am not interested in eating foods that I do not know, especially if they contain ingredients that I have never eaten before.” According to these three individual options, students were rated with a low, medium, or high level of food neophobia. The variable was thus coded as follows: 1 = low, 2 = medium, and 3 = high food neophobia.

### 2.6 Sustainability and health awareness

To measure student commitment to sustainability and health, we asked for consent for the following statements: “I prefer environmentally friendly food choices”; “I try to support sustainable developments”; and “I try to eat healthily.” Using a 4‐point Likert scale (1 = “strongly disagree,” 2 = “disagree,” 3 = “agree,” and 4 = “strongly agree”) respondents indicated the degree of agreement.

### 2.7 Willingness to accept insects as animal feed and human food after providing information

Participants were shown four statements that provided information about the nutritional and environmental benefits of using insects as food and feed to determine the role of the information in their willingness. The first two statements referred to insects as animal feed, while the last two statements referred to insects in human food. Using a 4‐point Likert scale (1 = “strongly disagree,” 2 = “disagree,” 3 = “agree,” and 4 = “strongly agree”), respondents indicated their degree of agreement in each statement.

### 2.8 Statistics and model

All statistics were performed using IBM SPSS statistics software package version 22 (IBM Corporation) for Windows. Distribution of frequency, mean scores, and standard deviations were determined from the results of the survey (*n* = 222). Cronbach's alpha tests were used to determine whether the constructs were homogeneous. When the alpha was higher than 0.7, it was assumed that the statements gave an indication of the topic measured values that are acceptable in exploratory research (Nunnally & Bernstein, 1994).

Willingness to accept insects as animal feed and willingness to accept insects in human food were considered the two dependent variables. Those variables were coded as *WillFood* and *WillFeed*. To explore the drivers of consumer acceptance, correlation analysis and multiple linear regression models were conducted. For this, the socio‐demographic and psychological factors, as well as the role of information, were used. These factors were coded as follows: nationality (*Nat*), age (*Age*), gender (*Gen*), diet (*Diet*), and food neophobia (*Neoph*). For the factors sustainable and healthy awareness, visual acceptance, and willingness after providing information, new parameters were created by merging items, utilizing the computed metric index value. The new parameters were also included in the model as variables as follows: For sustainable and healthy awareness, the new factor was coded as (*Awa*) (*α* = 0.8). For visual acceptance, the parameters were as follows: familiarity (*Fam*), novelty (*Nov*), and visibility (*Vis*) (*α* = 0.8). The new parameters for the role of information were grouped into information about insects as feed (*InfoFeed*) (*α* = 0.7) and information about insects as food (*InfoFood*) (*α* = 0.8).

## 3 RESULTS

### 3.1 Survey

Regarding the willingness to accept insects as animal feed, about 84% of students answered that they were willing (54 agreed and 30% strongly agreed) and 15% declared to be unwilling (10 disagreed and 5% strongly disagreed). In terms of willingness to include insects in human food, 48% of the students stated to be willing (40% agreed and 8% strongly agreed) and 49% were not (34% disagreed and 15% strongly disagreed). Uncertain participants represented about 3% in both cases (insects in human diets and insects in animal diets). The willingness to accept insects as animal feed was higher (*M* = 3.0 ± 0.8) than that of using insects in human food (*M* = 2.3 ± 0.9) (Table 2).

**Table 2:** Descriptive statistics of the survey items: mean scores, percentages and standard deviations (*n* = 222)

Survey items		
^a^Willingness to accept insects in animal feed and human food	Mean	*SD*
Would you agree to eating meat or eggs if the animals were raised with feed containing insect protein?	3.0	0.8
Would you agree to eating products containing insects (e.g., pancakes or pasta with insect powder)?	2.3	0.9
^b^Visual acceptability	Mean	*SD*
How likely would you eat the following foods?		
Pancakes with insect powder	2.8	0.9
Smoothie bowl with insect powder	2.5	0.9
Burger with insects	2.2	1.1
Insects covered in chocolate	2.1	1.0
Crickets: Insect protein snack	2.1	1.0
Insect lime‐and‐thyme balls	1.8	0.9
^c^Is there a reason you would not be willing to eat insect(powder) in your food?	%	
Disgust	43.8	
Lack of knowledge	18.4	
Other	16.0	
None of the above (willing to eat insects)	13.9	
Food safety	6.6	
Religion	1.3	
^c^Food neophobia	%	
I am open to trying new foods, but it should be close to what I already know (medium level).	49	
I often try new foods and I am curious about new products (low level).	41	
I am not interested in eating food I do not know, especially if it contains ingredients I have never eaten before (high level).	10	
Sustainable and healthy awareness	Mean	*SD*
I prefer environmentally friendly food choices.	3.0	0.7
I try to eat healthily.	3.0	0.8
I try to support sustainable developments.	2.9	0.7
^a^Willingness to accept insects in animal feed and human food after providing information	Mean	*SD*
If feeding animals with insects instead of soy would have health benefits for the animals’ immune system, I would consider eating products of these animals (meat, milk, eggs).	3.1	0.8
If feeding animals with insects instead of soy would have environmental benefits (e.g., less water and CO2 emissions) I would consider eating products of these animals (meat, milk, eggs).	3.1	0.8
If insect breeding has environmental benefits compared with meat production (e.g., less water and CO2 emissions), I would consider eating products made with them.	2.6	0.8
If adding insects to my diet would have health benefits for me (e.g., high in protein, good for digestion, boosting immune system), I would consider eating products made with them.	2.4	1.0

^a^ Item coded on a scale from 0 = “uncertain,” until 4 = “strongly agreed.”

^b^ Item coded on a scale from 1 = “highly unlikely,” until 4 = “highly likely.”

^c^ Item with single choice question.

Results from the socio‐demographic variables showed differences in the willingness to accept insects as animal feed between nationalities (*F*_(4,217)_ = 4.7, *p* < .01). The acceptance of Dutch students was higher (94%: 66% and 28% agree and strongly agree, respectively) than that of the German students (75%: 43% and 32% agree and strongly agree, respectively). Differences were also observed between diet groups (*F*_(4,217)_ = 6.9, *p* < .01). The highest percentage of students accepting insects as animal feed was observed among those who follow a VDF (87%: 56% and 31% agree and strongly agree, respectively) and flexitarians (88%: 65% and 23% agree and strongly agree, respectively). Of the 14 vegetarian respondents, 42% declared their acceptance (14% and 28% agree and strongly agree, respectively). Only two vegans participated in this study, and therefore, this group was excluded from the statistical analysis. For the variables age (*F*_(4,217)_ = 1.6, *p* = .17) and gender (*F*_(4,217)_ = 2.5, *p* = .06), no differences were observed for incorporating insects as animal feed. Similarly, when the answers about willingness to accept insect in human food were compared, no differences were observed in any of the socio‐demographic variables (nationality: *F*_(4,217)_ = 2.0, *p* = .09; age: *F*_(4,217)_ = 1.0, *p* = .38; gender: *F*_(4,217)_ = 1.5, *p* = .33; and diet: *F*_(4,217)_ = 1.6, *p* = .18).

In the visual test, it was observed that the food most liked was the pancakes (*M* = 2.8 ± 0.9; 42% and 26% likely and highly likely, respectively) followed by the smoothie bowl (*M* = 2.5 ± 0.9; 35% and 16% likely and highly likely, respectively), burgers (*M* = 2.2 ± 1.1; 22% and 16% likely and highly likely, respectively), crickets—protein snack (*M* = 2.1 ± 1.0; 22% and 12% likely and highly likely, respectively), and insects covered in chocolate (*M* = 2.1 ± 1.0; 22% and 12% likely and highly likely, respectively). The least liked were the lime‐and‐thyme balls (*M* = 1.8 ± 0.9; 16% and 6% likely and highly likely, respectively). In answer to the question: “Is there a reason why you would not be willing to eat insect (powder)?” participants declared mostly because of disgust (44%), followed by lack of knowledge (18%), other (16%), and finally food safety (3%). About 14% declared “none of the above,” indicating that they would be willing to eat insects.

According to the statement to measure food neophobia, 49%, 41%, and 10% of the respondents were categorized as having a medium, high, and low level of neophobia, respectively. From this, it can be said that the sampled population is at a medium level of neophobia, which implies that they are open to trying new foods, but they should be close to what they know.

Regarding awareness of sustainability and health, it was observed that participants are involved with these issues. About 79% of them prefer environmentally friendly food choices (*M* = 3.0 ± 0.7), 78% try to support sustainable developments (*M* = 2.9 ± 0.8), and 80% try to eat healthily (*M* = 3.0 ± 0.7).

When the participants were informed that feeding animals with insects has benefits for the animals’ immune system, 84% declared to be willing (50% agreed and 34% strongly agreed) and 15% declared to be unwilling (10% disagreed and 5% strongly disagreed) to eat products of these animals. Likewise, when participants were informed that feeding animals with insects also has environmental benefits, 82% were willing, while 17% claimed to be unwilling (12% disagreed and 5% strongly disagreed) to eat products of these animals (49% agreed and 33% strongly agreed). Uncertain participants represented 1% in both cases (benefits for the animals’ immune system and benefits for the environment). The scores obtained for the two statements were the same (*M* = 3.1 ± 0.8) and indicate that respondents are willing to include insects in animal diets because of such benefits.

When respondents were informed that insect breeding has environmental benefits compared with meat production, 60% were willing (45% agreed and 15% strongly agreed) and 39% were unwilling (30% disagreed and 9% strongly disagreed) to eat products made with insects. Similarly, 55% of them declared to be willing (40% agreed and 15% strongly agreed) and 42% declared to be unwilling (28% disagreed and 15% strongly disagreed) to eat products made with insects when they were informed that this would have health benefits for humans. Uncertain participants represented 1 and 3% of the total in both cases, respectively (environmental benefits and human benefits). The scores obtained for the two statements (*M* = 2.6 ± 0.8 for environmental benefits and *M* = 2.4 ± 1.0 for human benefits) indicate that respondents were more willing to include insects in their own food than when they were asked without mentioning the benefits (*M* = 2.3 ± 0.9).

### 3.2 Variable correlation

*WillFeed* had a significant positive correlation with *Nat* and *InfoFeed,* and had a significant negative correlation with *Diet* (Table 3). *WillFeed* did not have a significant correlation with *Age*, *Gen*, *Neoph,* or *Awa*. Since variables referring to direct insect consumption such as *Fam*, *Nov*, *Vis,* and *InfoFood* were considered not relevant for the explanation of *WillFeed*, they are not described. Similarly, *InfoFeed* was a variable considered not to be relevant for the explanation of *WillFood* since this refers to indirect insect consumption. *WillFood* had a positive correlation with *Fam*, *Nov*, *Vis,* and *InfoFood*, while *Neoph* had a negative correlation with it. *WillFood* did not have a significant correlation with *Nat*, *Age*, *Gen*, *Diet,* and *Awa*. Although low, for both cases (*WillFeed* and *WillFood*) the highest correlation was for the variables related to information on the benefits (*InfoFeed* and *InfoFood*).

**Table 3:** Correlation matrix

	Variables	1	2	3	4	5	6	7	8	9	10	11	12	13
1	WillFeed	1												
2	WillFood	0.37**	1											
3	Nat	0.14*	0.05	1										
4	Age	−0.05	−0.04	−0.10	1									
5	Gen	0.17	0.11	0.13*	−0.08	1								
6	Diet	−0.22**	−0.09	−0.21**	−0.05	−0.20**	1							
7	Neoph	−0.02	−0.27**	0.12	0.06	0.08	−0.21**	1						
8	Fam	0.17*	0.45**	−0.00	−0.06	0.12	−0.14*	−0.20**	1					
9	Nov	0.21**	0.45**	−0.13*	0.01	0.04	−0.05	−0.33**	0.62**	1				
10	Vis	0.06	0.33**	−0.12	−0.06	0.15*	−0.09	−0.20**	0.56**	0.49**	1			
11	Awa	−0.01	0.11	−0.15*	0.01	−0.25**	0.33**	−0.27**	−0.03	0.15*	0.09	1		
12	InfoFeed	0.39**	0.27**	0.02	−0.15*	0.10	−0.22**	−0.06	0.17*	0.24**	0.05	0.17*	1	
13	InfoFood	0.24**	0.46**	−0.12	−0.06	0.13	0.03	−0.21**	0.40**	0.43**	0.43**	0.20**	0.36**	1

Note

The table shows Pearson's correlation coefficients. Diagonal cells report the means. Gray cells indicate the correlation of the independent variables with the dependent variables.

Abbreviations: Age, age; Awa, healthy awareness; Diet, Diet; Fam, familiarity; Gen, gender; InfoFeed, information about insects as feed; InfoFood, information about insects as food; Nat, nationality; Neoph, food neophobia; Nov, novelty; Vis, visibility; WillFeed, willingness to accept insects as animal feed; WillFood, Willingness to accept insects in human food.

* Correlation is significant at the 0.05 level (2‐tailed).

** Correlation is significant at the 0.01 level (2‐tailed).

### 3.3 Multiple linear model

Multiple linear regression was performed for both dependent variables to check which predictors have the most significant influence on consumer acceptance (Table 4).

**Table 4:** Multiple regression models of the willingness to accept the use of insects in animal feed and human food

WillFeed	*R*	Variable	B	Std. error	*β*
Step I	.39	(Constant)	1.74**	0.22	
		InfoFeed	0.42**	0.07	0.39
Step II	.41	(Constant)	2.07**	0.26	
		InfoFeed	0.39**	0.07	0.36
		Diet	−0.18*	0.08	−0.14
WillFood					
Step I	.46	(Constant)	0.95**	0.19	
		InfoFood	0.54**	0.07	0.46
Step II	.54	(Constant)	0.47*	0.20	
		InfoFood	0.40**	0.07	0.33
		Fam	0.35**	0.07	0.31
Step III	.56	(Constant)	0.40*	0.21	
		InfoFood	0.35*	0.07	0.29
		Fam	0.23**	0.08	0.21
		Nov	0.22**	0.09	0.18
Step IV	.58	(Constant)	0.02	0.26	
		InfoFood	0.36**	0.07	0.31
		Fam	0.22**	0.08	0.19
		Nov	0.24**	0.09	0.21
		Nat	0.23*	0.11	0.12
Step V	.59	(Constant)	0.41	0.32	
		InfoFood	0.35**	0.07	0.29
		Fam	0.22**	0.08	0.19
		Nov	0.20**	0.09	0.17
		Nat	0.25**	0.11	0.13
		Neoph	−0.17**	0.08	−0.12

Abbreviations: Age, age; Awa, healthy awareness; Diet, Diet; Fam, familiarity; Gen, gender; InfoFeed, information about insects as feed; InfoFood, information about insects as food; Nat, nationality; Neoph, food neophobia; Nov, novelty; Vis, visibility; WillFeed, willingness to accept insects as animal feed; WillFood, Willingness to accept insects in human food.

*P ≤ 0.05, **P ≤ 0.01.

For *WillFeed*, two steps were performed taking as a positive predictor *InfoFeed* (*β* = 0.36; *p* < .01) and as a negative predictor *Diet* (*β* = −0.14; *p* < .01). *InfoFeed* had the strongest influence. The first step that included only *InfoFeed* explained a total variance of 40% (*F*_(1, 220)_ = 39.59; *p* < .01). The inclusion of *Diet* in the second step explained 41% of the variance (*F*_(2, 219)_ = 22.70; *p* < .01).

For the variable *WillFood*, five steps were performed taking *InfoFood* (*β* = 0.29; *p* < .01) as the predictor with the strongest influence. In addition, *Fam* (*β* = 0.19; *p* < .01), *Nov* (*β* = 0.17; *p* = .02), *Nat* (*β* = 0.13; *p* = .02) had a positive influence. A negative significant predictor was *Neoph* (*β* = −0.12; *p* = .03). Predictors were gradually inserted into the model in each step in the order mentioned. The first step of the multiple linear regression model explained a total variance of 46% (*F*_(2, 219)_ = 56.95; *p* < .01). The second (*F*_(2, 219)_ = 46.32; *p* < .01), third (*F*_(3, 218)_ = 33.72; *p* < .01), fourth (*F*_(4, 217)_ = 26.92; *p* < .01), and fifth (*F*_(5, 216)_ = 22.78; *p* < .01) step explained 54, 56, 57, and 58%, respectively.

## 4 DISCUSSION

### 4.1 Willingness to accept insects as animal feed and human food

In this study, respondents were willing to accept insects as animal feed (84%). Previous studies observed similar results. Kostecka et al. (2017) surveyed 210 consumers in Poland and claim positive attitudes concerning using insects to feed chicken, fish, cattle, and pigs, expressed by 58, 57, 42, and 47% acceptance, respectively. In its worldwide survey on consumer perception, PROteINSECT (2016) reports that 70% of respondents considered it acceptable to feed insect protein to farmed animals and, 66% were comfortable eating meat from a farmed animal fed on insect meal. Ferrer Llagostera et al. (2019) showed that the Spanish consumers were willing to pay a premium for fish fed with insects compared to fish produced with the current feeding systems. Likewise, in their research with citizens, stakeholders, and farmers in Belgium, Verbeke (2015) claims that attitudes toward using insects as animal feed were generally positive.

We also observed that the respondents were more willing to accept insects as animal feed than in their own diets. Less than half of them (48%) stated to be willing to eat products containing insects. Similar results have been previously observed. Laureati et al. (2016) reported that 53% of the Italian interviewees were ready to accept the incorporation of insects into animal diets. However, there was a drop to 21% of people who were willing to include insects in their human diet. Videbæk and Grunert (2020) found that both groups, Insect Feeders and Insect Opponents, displayed a positive attitude toward using insects as feed. Likewise, La Barbera et al. (2020) observed a negative relation between the variables concerning feeding animals with insects and the intention to consume insects. These results suggest that consumer acceptance of insects as animal feed is not a barrier to the development of the insect feed industry. However, this could indicate that consumers consider insects as suitable for animal feed, but not for human consumption. Individuals’ attitudes toward direct and indirect entomophagy may have relevant practical implications (La Barbera et al., 2020). The current use of insects as feed could potentially affect how consumers perceive insects in human nutrition. This could be an obstacle to greater acceptance of edible insects on the market (Videbæka & Grunerta, 2020). Further studies on this topic are required.

The willingness to consume insects directly still seems to be a barrier. In this study, the percentage of Dutch and German students who were willing to introduce insects in their food was higher than previous research conducted with other European consumers (Schösler et al., 2012; Tan et al., 2015; Vanhonacker et al., 2013; Verbeke, 2015). Differences in the willingness percentage between the previous and the present work may be due to the timing of the surveys. Some years ago, authors such as de Boer et al. (2013) concluded that there is room for a change to a diet with more environmentally friendly proteins in the Netherlands, as far as no insects are involved. Vanhonacker et al. (2013) reported that 5% of Belgians surveyed were willing to consume insects. Two years later, Verbeke (2015) reported that about 20% of Belgian respondents were willing or ready to integrate insects into their diet as food. An increase in the willingness to eat insects can also be observed in research conducted in the Netherlands (Fischer & Steenbekkers, 2018; Schösler et al., 2012; Tan et al., 2015). More recently, Kostecka et al. (2017) reported 37% acceptance of insect flour in Poland, and Lammers et al. (2019) observed that 42% of the German respondents were prepared to consume processed insect burgers.

The above may be the result of the efforts to spread information and advertising by European countries in recent years. Van Thielen et al. (2019) comment that since the introduction of insect‐based foods onto the market in 2016, most people in Belgium are aware that insect‐containing foods exist. In Germany, Berger et al. (2018) relate the positive impact of advertising strategies, such as popular media, TV documentaries and newspaper articles on consumers. Furthermore, in the last decade, many insect food companies are starting up in different European countries such as France, UK, Belgium, the Netherlands (La Barbera et al., 2018), and Germany (Orsi et al., 2019). This may have brought the idea of eating insects to the attention of consumers and shaped more favorable attitudes (Verbeke, 2015). For example, recently, Videbæk and Grunert (2020) found a segment of consumers that are willing to eat insects, motivated by the interest in edible insects. Likewise, Adámek et al. (2018) showed that protein and energy bars, enriched with cricket flour, were acceptable as a new type of food for consumers in the Czech Republic. These findings are consistent with the idea that an insect market may develop further in Western countries.

Insect consumption studies have shown that willingness to consume insects is influenced by socio‐demographic factors such as age (Barsics et al., 2017; Hartmann et al., 2015; Laureati et al., 2016; Tan, et al., 2016) or gender (Caparros Megido et al., 2014; Laureati et al., 2016; Menozzi et al., 2017a, 2017b; Sogari et al., 2017; Tan, et al., 2016). Another reason for a higher percentage of willingness to consume insects than in previous research may be because the population sampled was composed of young university students. Generally, it seems that the most reliable early adopters are young people with a high educational level (Cicatiello et al., 2016; Fischer & Steenbekkers, 2018). Previous studies have suggested that young, male individuals are more willing to eat insects than older people (Caparros Megido et al., 2014; Hartmann et al., 2015; Verbeke, 2015) and women (Barsics et al., 2017; Caparros Megido et al., 2014; Tan, et al., 2016). However, for age and gender, no differences were observed in the present study. Although our sample was limited to a small age group, these data could indicate that for 18‐ to 26‐year‐old German and Dutch students, gender does not affect willingness to accept insects as animal feed and human food.

Our research indicates that Dutch respondents were more willing to incorporate insects into animal diets than German ones. According to previous studies, the willingness to eat insects could be affected by nationality (Menozzi et al., 2017a, 2017b; Sogari, et al., 2019). The willingness is related to culture, and attitudes toward insect food vary across European regions (Deroy et al., 2015; Menozzi et al., 2017a, 2017b; Sogari, et al., 2019; Verneau et al., 2016). Nevertheless, there are no studies that relate to indirect consumption of insects with demographic variables. Additionally, the data from this study do not allow us to conclude whether this difference is due to cultural reasons between German and Dutch students.

A small percentage of the students indicated being vegetarian (6.3%) and vegan (0.9%), which corresponds to the expected normal Dutch (59% varied diet with meat, 37% flexitarians, 2% vegetarian, and <1% vegan) (Natuur & Milieu, 2019) and German (66% varied diet with meat, 34% flexitarian, 5% vegetarian and 2% vegan) (Nier, 2019) populations. As expected, vegetarians and vegans have a stronger aversion to eating insects (Orsi et al., 2019). The effects of these socio‐demographic and psychological factors, as well as the role of information, will be discussed separately below in the light of direct and indirect insect consumption.

### 4.2 Effect of factors and information on the willingness to accept insects as animal feed

Significant positive correlation of demographics (*Nat*) and information on the benefits of using insects as feed (*InfoFeed*), with the willingness to incorporate insects as animal feed (*WillFeed*) was observed. As noted earlier, we consider that our data do not allow us to support the demographic influence on the willingness to consume insects indirectly. Therefore we have discarded the relevance of correlation with the *Nat* variable. Additionally, *WillFeed* had a significant negative correlation with *Diet*. According to the multiple linear model, the most important predictors were *Diet* and *InfoFeed*. As observed, it was expected that diet (*Diet*) and information about benefits of feeding animals with insects (*InfoFeed*) had some effect on consumer acceptance.

The diet has an effect on consumer acceptance regarding entomophagy in previous studies (House, 2019). In the research by Elorinne et al. (2019), Finnish vegans were the least positive about insect consumption compared to omnivores and vegetarians. Moreover, the omnivores and nonvegan vegetarians considered entomophagy as a possibility for solving the world's food problems. However, this work, as well as the one of House (2019), addressed the direct consumption of insects. To our knowledge, this is the only research focused on vegetarians’ attitude toward insect‐based foods. Besides our present work, no studies have been carried out earlier relating diet to indirect consumption of insects. In order to understand the niche of insect feed regarding different food trends, it would be valuable to do an in‐depth study in the future on the psychology and acceptance of indirect entomophagy in different diet groups. An example could be eggs from insect‐fed hens for vegetarians. It has been proven that Black Soldier Fly larvae have a positive effect on laying hens (Star et al., 2020) and vegetarians could be aware of the environmental advantages.

As mentioned above, *InfoFeed* was an important predictor in the willingness to consume insects indirectly. This constitutes an important finding since all previous work has focused on predictors for direct consumption of insects. In accordance with our results, Bazoche et al. (2016) concluded that the information about the environmental impact seems to clearly increase the consumer's willingness to accept insect meal in animal feeding (aquaculture). Likewise, Szendrő et al. (2020) claimed that to increase consumer acceptance of meat products from animals reared on insect meal, consumers need to be made aware of the various benefits of insect meal in animal feed. In their survey, Hungarian respondents gave 5.11 points for the meat of free‐range animals, but significantly fewer points (3.69) for the meat of animals that had consumed insect meal. An interesting point that the authors discuss is that free‐range is associated with animal welfare, but free‐range feed naturally on insects. Because consumers tend to have little knowledge about feeds and their impact on the environment, they generally have no strong opinions about the subject. More information may increase awareness and the likelihood that people will accept insect‐based feeds (Popoff et al., 2017).

### 4.3 Influence of factors and information on willingness to accept insects in human food

Significant positive correlation of the factors related to visual acceptance of different insect‐based food (*Fam*, *Nov*, *Vis*) and information on the benefits of using insects in food (*InfoFood*) with the willingness to incorporate insects in human food (*WillFood*) was observed. By contrast, neophobia (*Neoph*) had a negative correlation with it. In accordance with this, the multiple linear regression showed *Fam*, *Nov*, *Nat* and *InfoFood* as positive, and *Neoph* as negative in terms of most important predictors of influence.

The variables *Fam*, *Nov*, *Vis* represent familiarity, novelty and visibility, respectively. These variables were a strong predictor of the acceptance of eating insects. In line with our theoretical model, respondents showed more willingness to try familiar insect foods such as the pancakes. Familiarity (*Fam*) turns out to be an essential supporter to consumption in European countries, and it is a driver for positive attitudes toward edible insects (Caparros Megido et al., 2016; Piha et al., 2018; Tan et al., 2015; Verneau et al., 2016). On the other hand, a controversial result was that the majority of students did not accept the hamburgers in the visual test, even though it is a well‐known food that is said to be well suited for the introduction of insects (Van Thielen et al., 2019). This may be related to the unwillingness to substitute real meat (burger) for an alternative product. Hartmann and Siegrist (2017) reviewed 38 articles about consumer perception regarding sustainable protein. One of their main conclusions was that the willingness to change meat consumption behavior in terms of reducing or substituting meat (e.g., by eating insects or meat substitutes) is surprisingly low in Europe.

The visual appearance of foods could be a critical factor for their acceptance. The participants were more likely to accept the foods when the insects were “invisibly” incorporated in a recipe than when they were visible (*Vis*). Several studies have evaluated consumer acceptance of visible and invisible insects as ingredients (Caparros Megido et al., 2016; Schösler et al., 2012; Tan et al., 2015; Tan, et al., 2016; Verneau et al., 2016). Their main conclusion was that visible insects were rejected more often than meals in which the insects were not visible. Lensvelt and Steenbekkers (2014) suggested that insects could be mixed into other dishes or products to lower the barrier. In our study, however, we found that a third of the respondents were willing to eat cricket protein snacks and chocolate‐covered insects (visible insect products). Therefore, factors such as curiosity can play an important role in some consumer groups. Studies in Germany, such as Orsi et al. (2019), obtained negative results in this regard. Their samples indicated a rather low level of curiosity to try any of the products. By contrast, Sogari (2015) concluded that in Italy, curiosity is one of the most important factors in motivating the consumption of insects in the future. As reported for Menozzi et al., 2017a; Menozzi et al., 2017b, we could highlight the positive role of curiosity, especially among young consumers.

On the other hand, flavoring components of food also play a role in their acceptance. It has been shown that ingredients (Tan, et al., 2016) and preparation methods (Caparros Megido et al., 2014) that are perceived as a cultural novelty (*Nov*) affect consumer acceptance. In the present study, the lowest acceptance was observed for the lime‐and‐thyme insect balls (33%). In addition to the insects, the “lime‐and‐thyme” taste, with which the food is described, could have led to rejection. As in the case of previous research in other European countries (Caparros Megido et al., 2016; Tan et al., 2015; Van Thielen et al., 2019), we suggest that for German and Dutch young consumers the food industry should focus on processed insect‐based foods with familiar recipes, which would presumably lead to a higher willingness to eat.

According to our theoretical framework, food neophobia had a negative influence on consumer acceptance. That means that the lower the values of neophobia, the greater the willingness of students to consume insects. Previous studies have discussed the influence of food neophobia on the willingness to try insects, claiming that it is one of the most important factors to predict it (Hartmann et al., 2015; Hartmann & Siegrist, 2016; Sogari, et al., 2019; Verbeke, 2015; Verneau et al., 2016). This finding is a clear reflection on the role of familiar, novel and visual components of foods previously discussed. Nevertheless, recent studies found that food neophobia is no longer the key barrier to insect consumption (Fischer & Steenbekkers, 2018; Schlup & Brunner, 2018). As edible insects are becoming more familiar to the consumers, as they see them in supermarkets or hear about them in the news, the food neophobia could be replaced by an interest in the products instead (Videbæk & Grunert, 2020). Perhaps, nowadays, a better determining factor for edible insect acceptance may be the information that consumers have or receive about the benefits of eating such food.

As mentioned above, our data showed that the information about the benefits of insects as food (*InfoFood*) was an essential predictor of willingness to incorporate insects into human diets. This result highlights the role of information to overcome the reluctance to eat insects. Indeed, studies have indicated that promoting information about the benefits of edible insects (environmental and health aspects) could improve the consumer's willingness in different European countries (Barsics et al., 2017; Laureati et al., 2016; Lensvelt & Steenbekkers, 2014; Lombardi et al., 2019; Orsi et al., 2019; Sogari, et al., 2019; Verbeke, 2015; Verneau et al., 2016; Verneau et al., 2016).

### 4.4 Sustainable and healthy awareness

In our research, the majority of the participants declared that they made environmentally friendly food choices, supported sustainable developments and tried to eat healthily. We highlight this result because this factor was not a significant predictor for the willingness, despite the strong sustainability and healthy awareness (*Awa*). Studies state that environmental awareness is a factor that positively influences consumer acceptance (Hartmann & Siegrist, 2018; Kostecka et al., 2017; Lensvelt & Steenbekkers, 2014; Menozzi et al., 2017a, 2017b; Tan et al., 2015; Verbeke, 2015). However, we agree with the idea that it is not enough to stimulate the consumption of edible insects. For example, Laureati et al. (2016) reported that Italian consumers who declared themselves to behave sustainably indicated their uncertainty and disagreement regarding the possible use of insects in animal and human diets. Likewise, in their cross‐cultural study between Germany and China, Hartmann et al. (2015), showed that German consumers are not motivated by favorable sustainability attributes. Lammers et al. (2019) showed that for German consumers’ sustainability consciousness was also not a significant predictor of the willingness to consume insects. In the same way, regarding health consciousness, Hartmann et al. (2015) showed in their cross‐cultural study that Germans who focus on a healthy diet did not show the likelihood to consume whole insects.

### 4.5 Implications

Our results may be related to a lack of knowledge and/or belief in actual benefits of entomophagy on the environment and/or health. According to our data, the second reason our participants would not be willing to eat insects is a lack of knowledge, after disgust. Therefore, it is essential to create strategies that enable consumers to be educated about edible insects. Some organizations currently lobby in the Netherlands and Germany for the use of insects as a human food. Some stores have already included mainly locusts and mealworms in their products. The insects are marketed as a delicacy and can be purchased in internet shops (Schösler et al., 2012). However, the demand for insect products has to be created by intensifying education. Transparency in the food chain and reliable explanations about sustainability and health are key to this strategy. Thereby, consumers could become familiar with the practice and begin to adopt it into their daily diets. For instance, Sogari, et al., 2019 suggest that the adoption of information campaigns on sustainability and health issues of such products could increase consumers’ propensity to introduce insects into their diets. New studies should focus on dissemination of information. The potential of the currently researched insects is promising for both the environmental benefit in production and the health benefit in nutrient profile. Both parameters can be strong arguments for convincing the European consumer.

### 4.6 Limitations

The biggest limitation of this study was the use of a sample only represented by university students, which strongly restricts our capacity to generalize results for the German or Dutch population. Likewise, the sample size could have been greater in order to better represent minorities such as vegetarians and vegans, and cold have included more broadly potential consumers.

## 5 CONCLUSION

We present here the results of an exploratory research which allows some insights into consumer acceptance regarding insects in feed and food. These insights may be useful to address future studies, for instance, by focusing on a small segment of possible early young adopters and consequently addressing communication strategies in this market segment.

This study found that young German and Dutch university students are more willing to accept insects as animal feed than in their own diets. The acceptance among German and Dutch students toward insects in feed and food seems to be driven by issues similar to those in other European countries, such as familiarity with food, visibility of the insects, and knowledge about the benefits of entomophagy. These factors were the most important predictors of willingness to incorporate insects into human food and animal feed for the sampled population. The effect of the information on the willingness to consume insects indirectly constitutes an important finding, since most of the previous studies have focused on predictors for direct consumption. We highlight the need for more studies addressing consumer acceptance of insects as animal feed.

Western countries frequently do not accept insect consumption, mainly due to their lack of familiarity with entomophagy. Our results confirm the idea that rather than invest efforts in reducing neophobia and disgust, it could be more effective to aim at those that increase the level of familiarity with the dishes and interest about entomophagy. Likewise, our data support the need to inform and educate consumers about the environmental and health benefits of entomophagy.

## CONFLICT OF INTEREST

There are no conflicts of interest.

## ETHICAL APPROVAL

Ethical approval statement described in Appendix S2.

## Supporting information

App S1

App S2

App S3

## Data Availability

All data generated or analyzed during this study are included in this published article (and its supplementary information files) (Appendix S3).
