# Supplementary material for: Consumer acceptance among Dutch and German students of insects in feed and food
Source: Food Sci Nutr. 2020 Dec 1;9(1):414–28. doi: 10.1002/fsn3.2006 (PMC7802571; doi:10.1002/fsn3.2006)
Supplement: Supplementary file 1 — App S1 [file FSN3-9-414-s001.pdf]

Survey about acceptance of new food products

Fontys is partner in an EU financed project to investigate opportunities for alternative protein sources. This survey is conducted to find out about the motives of eating new food products.

By filling in the survey you support our research project. The results will help to develop innovative products which are suitable to feed the world. Thank you very much for your support!

Demographics:

☐ German ☐ Dutch

☐ International: ______________________ (please state your nationality)

Age:

☐ <18 ☐ 24-26
☐ 18-23 ☐ >26

Gender:

☐ Female ☐ Male ☐ Diverse

Study Year:

☐ 1st ☐ 3rd
☐ 2nd ☐ 4th and >4th

Study course: ____

1. Would you agree to eating meat or eggs if the animals were raised with feed containing insect protein?

Strongly Disagree	Disagree	Agree	Strongly Agree
(1)	(2)	(3)	(4)

2. Would you agree to eating products containing insects (e.g. pancakes or pasta with insect powder)?

Strongly Disagree	Disagree	Agree	Strongly Agree
(1)	(2)	(3)	(4)

3. On a scale from 1 – 5 how likely would you eat the following foods?
(1: highly unlikely, 2: unlikely, 3: likely - 4: highly likely)

1 (Pancakes)	1	2	3	4	4 (Smoothie bowl)	1	2	3	4
2 (Burger)	1	2	3	4	5 (Chocolate)	1	2	3	4
3 (Insect lime balls)	1	2	3	4	6 (Protein snack)	1	2	3	4

Is there a reason you would not be willing to eat insect(powder) in your food?
(more than one answer possible)

- ☐ lack of knowledge
- ☐ religion
- ☐ disgust
- ☐ food safety
- ☐ none of the above (willing to eat insects)
- ☐ other: ____

4. Which statement fits best to you? (only one)

- ☐ I often try new foods and I am curious about new products
- ☐ I am open to try new foods, but it should be close to what I already know.
- ☐ I am not interested in eating food I do not know, especially if it contains ingredients I have never eaten before

For the following statements indicate your level of agreement

5. I prefer environmentally friendly food choices.

Strongly Disagree	Disagree	Agree	Strongly Agree
(1)	(2)	(3)	(4)

6. I try to support sustainable developments.

Strongly Disagree	Disagree	Agree	Strongly Agree
(1)	(2)	(3)	(4)

7. I try to eat healthy.

Strongly Disagree	Disagree	Agree	Strongly Agree
(1)	(2)	(3)	(4)

8. If **feeding animals** with insects instead of soy would have health benefits for the animals´ immune system, I would consider eating products of these animals. (meat, milk, eggs)

Strongly Disagree	Disagree	Agree	Strongly Agree
(1)	(2)	(3)	(4)

9. If **feeding animals** with insects instead of soy would have environmental benefits (e.g. less water and CO2 emissions) I would consider eating products of these animals (meat, milk, eggs).

Strongly Disagree	Disagree	Agree	Strongly Agree
(1)	(2)	(3)	(4)

10. If **insect breeding** had environmental benefits compared with meat production (e.g. less water and CO2 emissions), I would consider eating products made with them.

Strongly Disagree	Disagree	Agree	Strongly Agree
(1)	(2)	(3)	(4)

11. If adding insects to **my diet** would have health benefits for me (e.g. high in protein, good for digestion, boosting immune system), I would consider the consumption.

Strongly Disagree	Disagree	Agree	Strongly Agree
(1)	(2)	(3)	(4)

12. Which diet are you on? (How do you eat)

- ☐ varied diet with meat and/or fish
- ☐ flexitarian
- ☐ vegetarian
- ☐ vegan
