# Supplementary material for: Consumer acceptance among Dutch and German students of insects in feed and food
Source: Food Sci Nutr. 2020 Dec 1;9(1):414–28. doi: 10.1002/fsn3.2006 (PMC7802571; doi:10.1002/fsn3.2006)
Supplement: Supplementary file 2 — App S2 [file FSN3-9-414-s002.pdf]

Fontys Hogescholen

Fontys Commissie Ethiek van Onderzoek voor het domein Mens en Maatschappij

Datum:	14-10-2020
Adviesnr.:	*Naranjo-Guevara14102020 FCEO, behandeld op 14 oktober 2020*
Onderwerp	advies n.a.v. beoordeling ethische factor onderzoeks- artikel
Onderzoeksvoorstel:	Consumer acceptance among Dutch and German students towards insects in feed and food
Behandeling:	De Fontys Commissie Ethiek van Onderzoek (FCEO) heeft na ontvangst van de toegezonden stukken de vragenlijst en het onderzoeks-artikel beoordeeld op 14 oktober 2020.
Inhoudelijke reactie:	Geachte Mevr. Naranjo, beste Natalia,

De Fontys Commissie Ethiek van Onderzoek (FCEO) heeft het onderzoeks-artikel en vragenlijst behandeld.

Het onderzoek heeft als onderwerp: Consumer acceptance among Dutch and German students towards insects in feed and food

Vanwege hun milieu- en voedingsvoordelen lijkt de consumptie van insecten een van de oplossingen te zijn om de groeiende menselijke bevolking te voeden. Ondanks de toenemende belangstelling voor het gebruik van insecten als voedsel en diervoeder, lijkt de acceptatie van de consument echter het belangrijkste obstakel voor een succesvolle implementatie in westerse landen. Bij het zoeken naar strategieën om het proces om insecten te accepteren als onderdeel van diervoeder en menselijke voeding te versnellen, zijn de factoren onderzocht die de acceptatie van de consument beïnvloeden bij een groep jonge universiteitsstudenten uit Duitsland en Nederland. Er is een enquête gehouden onder een steekproef van 222 deelnemers. Socio-demografische en psychologische factoren werden vastgesteld op basis van een theoretische review. De invloed van die factoren op de bereidheid om insecten te accepteren in diervoeding en menselijke voeding werd geanalyseerd door correlaties en meerdere lineaire regressies. De belangrijkste resultaten laten zien dat er meer bereidheid is om insecten in diervoeding op te nemen dan in menselijke voeding. De acceptatie werd gedreven door visuele aspecten (bekendheid, nieuwheid en zichtbaarheid van insecten), huidige voeding en informatie over de voordelen van entomofagie. Het effect van de informatie op de bereidheid is een belangrijke bevinding van deze studie, met name voor het gebruik van insecten in diervoeding, aangezien de meeste eerdere studies gericht waren op het gebruik van insecten als menselijke voeding. Men concludeert dat effectieve inspanningen om entomofagie te implementeren de bekendheid van het insectenvoer kunnen vergroten en de (of goed opgeleide) consumenten kunnen informeren over de voordelen ervan.

Op basis van de aangeleverde informatie kan worden aangegeven dat het onderzoek naar alle waarschijnlijkheid niet onder de reikwijdte van de Wet Medisch Wetenschappelijk Onderzoek met mensen (WMO) viel. Het onderzoek betrof geen medisch-wetenschappelijk onderzoek. De opzet van het onderzoek en de uitvoering van het onderzoek vallen binnen de kaders van de Fontys Commissie Ethiek van onderzoek, daarom wordt de opzet van het onderzoek zoals beschreven in het artikel goedgekeurd, de commissie heeft geen verbetersuggesties voor de gekozen aanpak. Het geheel is methodologisch verantwoord en correct uitgevoerd..
We vertrouwen erop U hiermee voldoende geïnformeerd te hebben.

Met vriendelijke groeten,

Mevr. V. Lenselink, secretaris Fontys Commissie Ethiek van Onderzoek

| t: +318850-70586 • m: +31622564109 • e : v.lenselink@fontys.nl
