# Supplementary material for: Consumer acceptance among Dutch and German students of insects in feed and food
Source: Food Sci Nutr. 2020 Dec 1;9(1):414–28. doi: 10.1002/fsn3.2006 (PMC7802571; doi:10.1002/fsn3.2006)
Supplement: Supplementary file 3 — App S3 [file FSN3-9-414-s003.pdf]

Demograpł	Age	Gender	Year	Course	AnimalsInF	AnimalsInF	Pancakes	Burger
2,00	2,00	2,00	1,00	1,00	3,00	2,00	3,00	1,00
2,00	2,00	1,00	2,00	1,00	3,00	1,00	1,00	1,00
2,00	2,00	1,00	2,00	1,00	4,00	2,00	4,00	1,00
2,00	2,00	1,00	2,00	1,00	3,00	3,00	3,00	2,00
2,00	2,00	1,00	1,00	3,00	2,00	3,00	3,00	1,00
2,00	2,00	2,00	1,00	1,00	1,00	1,00	4,00	4,00
2,00	2,00	2,00	1,00	1,00	4,00	3,00	4,00	2,00
1,00	2,00	1,00	4,00	1,00	4,00	4,00	4,00	4,00
1,00	2,00	1,00	1,00	4,00	2,00	3,00	3,00	2,00
2,00	2,00	2,00	1,00	4,00	3,00	3,00	4,00	2,00
2,00	1,00	3,00	1,00	4,00	3,00	2,00	3,00	1,00
2,00	2,00	2,00	2,00	5,00	4,00	3,00	2,00	1,00
2,00	2,00	2,00	2,00	5,00	4,00	3,00	4,00	2,00
2,00	2,00	2,00	1,00	4,00	3,00	3,00	2,00	2,00
2,00	2,00	2,00	1,00	4,00	3,00	3,00	2,00	4,00
2,00	2,00	1,00	1,00	4,00	3,00	2,00	2,00	2,00
2,00	2,00	2,00	1,00	4,00	3,00	2,00	3,00	2,00
2,00	1,00	2,00	1,00	4,00	3,00	2,00	2,00	1,00
2,00	2,00	2,00	1,00	4,00	3,00	2,00	3,00	2,00
2,00	2,00	2,00	1,00	4,00	3,00	3,00	4,00	2,00
2,00	2,00	2,00	1,00	4,00	3,00	3,00	4,00	2,00
2,00	2,00	2,00	1,00	4,00	3,00	2,00	4,00	3,00
2,00	1,00	2,00	1,00	4,00	4,00	3,00	4,00	2,00
2,00	2,00	2,00	1,00	4,00	4,00	4,00	4,00	4,00
2,00	2,00	1,00	1,00	4,00	3,00	1,00	4,00	2,00
2,00	2,00	2,00	1,00	4,00	4,00	4,00	4,00	4,00
2,00	2,00	0,00	1,00	4,00	3,00	3,00	4,00	1,00
2,00	1,00	2,00	1,00	4,00	3,00	0,00	3,00	2,00
2,00	2,00	2,00	1,00	4,00	4,00	3,00	3,00	3,00
2,00	1,00	2,00	1,00	4,00	3,00	3,00	3,00	2,00
2,00	2,00	2,00	1,00	4,00	3,00	3,00	3,00	2,00
2,00	2,00	1,00	2,00	5,00	3,00	3,00	4,00	3,00
2,00	2,00	2,00	2,00	6,00	3,00	2,00	3,00	1,00
2,00	2,00	2,00	2,00	6,00	4,00	2,00	2,00	1,00
1,00	2,00	1,00	2,00	2,00	1,00	3,00	1,00	3,00
1,00	2,00	1,00	2,00	2,00	4,00	3,00	4,00	3,00
1,00	2,00	2,00	1,00	2,00	4,00	2,00	3,00	2,00
1,00	2,00	2,00	1,00	2,00	4,00	0,00	3,00	1,00
1,00	2,00	1,00	1,00	2,00	4,00	3,00	3,00	1,00
1,00	2,00	2,00	1,00	2,00	3,00	1,00	2,00	2,00
1,00	2,00	2,00	1,00	2,00	3,00	3,00	2,00	1,00
1,00	2,00	2,00	1,00	4,00	0,00	2,00	2,00	2,00
1,00	2,00	2,00	1,00	2,00	2,00	1,00	3,00	3,00
1,00	2,00	1,00	1,00	2,00	0,00	3,00	4,00	4,00
1,00	2,00	2,00	1,00	2,00	4,00	3,00	4,00	4,00
1,00	2,00	2,00	1,00	2,00	4,00	2,00	1,00	1,00

1,00	2,00	1,00	2,00	2,00	3,00	3,00	3,00	3,00
1,00	2,00	2,00	1,00	2,00	4,00	3,00	3,00	2,00
1,00	2,00	1,00	1,00	2,00	3,00	3,00	3,00	2,00
1,00	2,00	2,00	1,00	2,00	3,00	3,00	3,00	3,00
2,00	2,00	1,00	1,00	2,00	3,00	2,00	1,00	1,00
2,00	2,00	1,00	1,00	2,00	4,00	1,00	3,00	1,00
2,00	2,00	2,00	1,00	2,00	3,00	2,00	3,00	4,00
2,00	1,00	2,00	1,00	2,00	3,00	3,00	4,00	4,00
2,00	2,00	2,00	1,00	2,00	3,00	2,00	1,00	1,00
2,00	2,00	2,00	1,00	2,00	3,00	3,00	1,00	1,00
2,00	2,00	2,00	1,00	2,00	3,00	2,00	3,00	3,00
2,00	2,00	2,00	1,00	2,00	3,00	3,00	3,00	1,00
1,00	2,00	1,00	1,00	2,00	2,00	1,00	2,00	1,00
1,00	2,00	2,00	1,00	2,00	2,00	2,00	4,00	4,00
1,00	2,00	2,00	1,00	2,00	3,00	3,00	3,00	3,00
1,00	2,00	1,00	1,00	2,00	1,00	1,00	4,00	2,00
1,00	2,00	1,00	1,00	2,00	1,00	1,00	2,00	1,00
1,00	2,00	2,00	1,00	2,00	4,00	3,00	3,00	3,00
1,00	2,00	2,00	0,00	2,00	4,00	4,00	3,00	4,00
1,00	2,00	2,00	1,00	2,00	2,00	2,00	3,00	3,00
1,00	2,00	1,00	1,00	2,00	2,00	3,00	3,00	1,00
1,00	2,00	2,00	1,00	2,00	2,00	1,00	2,00	1,00
1,00	2,00	2,00	1,00	2,00	3,00	3,00	3,00	2,00
1,00	2,00	1,00	1,00	2,00	4,00	3,00	3,00	3,00
1,00	2,00	2,00	0,00	2,00	3,00	2,00	1,00	1,00
1,00	2,00	1,00	1,00	2,00	3,00	2,00	3,00	1,00
1,00	2,00	2,00	1,00	2,00	3,00	1,00	1,00	1,00
1,00	2,00	2,00	1,00	2,00	3,00	3,00	4,00	2,00
1,00	2,00	1,00	1,00	2,00	4,00	2,00	3,00	1,00
1,00	2,00	2,00	2,00	2,00	2,00	1,00	1,00	1,00
2,00	2,00	2,00	2,00	7,00	4,00	3,00	4,00	4,00
2,00	3,00	1,00	3,00	7,00	3,00	3,00	3,00	2,00
1,00	2,00	1,00	1,00	7,00	1,00	1,00	1,00	1,00
1,00	2,00	2,00	3,00	8,00	3,00	1,00	4,00	1,00
1,00	2,00	1,00	2,00	7,00	3,00	2,00	3,00	1,00
1,00	2,00	1,00	4,00	3,00	3,00	2,00	2,00	2,00
1,00	2,00	1,00	2,00	7,00	3,00	3,00	3,00	1,00
1,00	2,00	2,00	3,00	3,00	3,00	2,00	2,00	1,00
1,00	2,00	2,00	2,00	7,00	4,00	3,00	4,00	3,00
1,00	2,00	1,00	2,00	7,00	4,00	3,00	4,00	1,00
1,00	2,00	2,00	2,00	7,00	3,00	3,00	3,00	1,00
1,00	2,00	1,00	1,00	7,00	3,00	3,00	4,00	3,00
1,00	2,00	1,00	1,00	7,00	3,00	3,00	4,00	4,00
1,00	2,00	2,00	2,00	7,00	3,00	1,00	3,00	2,00
1,00	3,00	2,00	2,00	7,00	4,00	3,00	4,00	3,00
1,00	2,00	2,00	2,00	7,00	4,00	2,00	3,00	1,00
2,00	2,00	1,00	2,00	2,00	4,00	1,00	1,00	1,00

2,00	2,00	2,00	2,00	2,00	3,00	2,00	2,00	3,00
2,00	2,00	2,00	2,00	2,00	3,00	2,00	3,00	2,00
2,00	2,00	2,00	2,00	0,00	0,00	0,00	3,00	2,00
2,00	2,00	2,00	2,00	2,00	4,00	3,00	4,00	0,00
2,00	2,00	2,00	2,00	2,00	4,00	3,00	4,00	2,00
2,00	3,00	2,00	2,00	2,00	3,00	3,00	4,00	4,00
1,00	4,00	3,00	4,00	2,00	2,00	1,00	2,00	3,00
1,00	2,00	2,00	2,00	2,00	4,00	4,00	4,00	4,00
1,00	2,00	1,00	2,00	2,00	4,00	0,00	3,00	3,00
1,00	2,00	2,00	2,00	2,00	4,00	4,00	4,00	4,00
1,00	3,00	2,00	2,00	2,00	3,00	3,00	4,00	3,00
1,00	2,00	2,00	2,00	2,00	3,00	2,00	3,00	3,00
1,00	2,00	2,00	2,00	2,00	3,00	3,00	3,00	4,00
1,00	2,00	2,00	2,00	2,00	3,00	2,00	2,00	3,00
1,00	2,00	2,00	2,00	2,00	3,00	2,00	2,00	2,00
1,00	2,00	2,00	4,00	2,00	3,00	0,00	3,00	2,00
1,00	2,00	2,00	2,00	2,00	3,00	2,00	3,00	2,00
1,00	2,00	1,00	2,00	2,00	3,00	1,00	3,00	1,00
1,00	2,00	1,00	4,00	2,00	3,00	3,00	3,00	3,00
1,00	2,00	1,00	2,00	2,00	2,00	3,00	3,00	2,00
1,00	2,00	1,00	2,00	2,00	1,00	1,00	3,00	4,00
1,00	2,00	1,00	2,00	2,00	4,00	1,00	3,00	2,00
1,00	2,00	1,00	2,00	2,00	1,00	1,00	3,00	1,00
1,00	2,00	1,00	2,00	2,00	4,00	4,00	4,00	3,00
2,00	2,00	1,00	1,00	5,00	3,00	0,00	4,00	3,00
2,00	4,00	2,00	4,00	6,00	4,00	2,00	1,00	4,00
2,00	2,00	1,00	2,00	2,00	3,00	3,00	3,00	1,00
2,00	2,00	2,00	2,00	2,00	3,00	1,00	3,00	1,00
2,00	2,00	2,00	3,00	2,00	3,00	4,00	3,00	4,00
2,00	2,00	1,00	2,00	9,00	3,00	2,00	1,00	1,00
2,00	2,00	1,00	2,00	9,00	3,00	2,00	1,00	1,00
2,00	2,00	1,00	2,00	9,00	3,00	2,00	3,00	2,00
2,00	2,00	1,00	2,00	9,00	4,00	1,00	3,00	1,00
1,00	2,00	2,00	2,00	0,00	3,00	4,00	2,00	3,00
1,00	2,00	2,00	2,00	2,00	3,00	1,00	2,00	1,00
1,00	2,00	2,00	2,00	3,00	1,00	2,00	2,00	1,00
1,00	2,00	2,00	2,00	2,00	3,00	3,00	2,00	3,00
1,00	2,00	1,00	2,00	2,00	2,00	2,00	1,00	1,00
1,00	2,00	1,00	2,00	2,00	3,00	2,00	2,00	2,00
1,00	2,00	2,00	2,00	2,00	3,00	2,00	3,00	2,00
1,00	2,00	1,00	2,00	1,00	4,00	4,00	4,00	4,00
1,00	2,00	1,00	2,00	3,00	3,00	2,00	1,00	1,00
1,00	2,00	2,00	1,00	3,00	3,00	2,00	3,00	3,00
1,00	2,00	2,00	2,00	3,00	2,00	3,00	2,00	3,00
1,00	2,00	1,00	1,00	2,00	3,00	3,00	2,00	3,00
1,00	4,00	1,00	2,00	4,00	2,00	2,00	4,00	4,00
1,00	2,00	2,00	1,00	3,00	3,00	3,00	3,00	2,00

1,00	2,00	2,00	1,00	3,00	3,00	2,00	2,00	3,00
1,00	2,00	3,00	1,00	3,00	4,00	3,00	1,00	1,00
1,00	2,00	1,00	1,00	3,00	2,00	1,00	2,00	1,00
1,00	2,00	2,00	1,00	3,00	4,00	4,00	3,00	4,00
1,00	2,00	2,00	1,00	3,00	3,00	3,00	2,00	3,00
1,00	2,00	2,00	1,00	3,00	3,00	2,00	3,00	2,00
1,00	2,00	2,00	1,00	3,00	3,00	2,00	2,00	2,00
1,00	2,00	2,00	1,00	3,00	3,00	3,00	4,00	4,00
1,00	4,00	1,00	4,00	2,00	3,00	2,00	1,00	1,00
2,00	1,00	1,00	1,00	9,00	3,00	2,00	3,00	1,00
2,00	2,00	2,00	2,00	4,00	3,00	2,00	3,00	2,00
2,00	2,00	2,00	2,00	4,00	3,00	3,00	3,00	3,00
2,00	2,00	2,00	2,00	4,00	3,00	3,00	4,00	3,00
2,00	3,00	2,00	2,00	4,00	3,00	3,00	3,00	2,00
2,00	2,00	1,00	2,00	4,00	3,00	2,00	4,00	1,00
2,00	2,00	2,00	2,00	5,00	3,00	3,00	4,00	3,00
2,00	2,00	2,00	2,00	5,00	3,00	2,00	3,00	3,00
2,00	2,00	1,00	2,00	4,00	3,00	2,00	3,00	3,00
2,00	3,00	2,00	4,00	1,00	3,00	2,00	1,00	1,00
1,00	2,00	1,00	1,00	3,00	4,00	4,00	4,00	3,00
1,00	2,00	2,00	3,00	2,00	4,00	4,00	4,00	4,00
1,00	2,00	2,00	4,00	2,00	4,00	4,00	3,00	3,00
1,00	2,00	2,00	4,00	2,00	4,00	3,00	4,00	4,00
1,00	2,00	2,00	2,00	2,00	4,00	4,00	4,00	4,00
1,00	2,00	1,00	2,00	2,00	4,00	3,00	3,00	3,00
2,00	2,00	1,00	1,00	8,00	3,00	2,00	3,00	1,00
1,00	2,00	1,00	2,00	2,00	1,00	2,00	2,00	1,00
1,00	2,00	1,00	2,00	2,00	4,00	1,00	2,00	1,00
1,00	2,00	2,00	1,00	0,00	3,00	3,00	2,00	4,00
1,00	2,00	2,00	2,00	7,00	3,00	2,00	2,00	1,00
1,00	2,00	2,00	1,00	8,00	3,00	2,00	3,00	2,00
1,00	2,00	1,00	1,00	7,00	1,00	1,00	2,00	1,00
1,00	2,00	2,00	1,00	7,00	4,00	3,00	3,00	2,00
1,00	2,00	2,00	1,00	7,00	4,00	2,00	3,00	2,00
1,00	2,00	2,00	1,00	7,00	4,00	1,00	1,00	1,00
1,00	2,00	2,00	1,00	7,00	4,00	3,00	2,00	2,00
1,00	2,00	2,00	1,00	7,00	3,00	2,00	3,00	4,00
1,00	2,00	2,00	1,00	7,00	3,00	2,00	2,00	2,00
1,00	4,00	0,00	2,00	7,00	3,00	3,00	2,00	2,00
1,00	2,00	2,00	1,00	7,00	3,00	2,00	4,00	4,00
1,00	2,00	2,00	1,00	7,00	3,00	3,00	3,00	2,00
1,00	2,00	2,00	0,00	7,00	4,00	4,00	3,00	1,00
2,00	2,00	2,00	2,00	4,00	3,00	3,00	3,00	3,00
2,00	2,00	2,00	2,00	4,00	3,00	2,00	3,00	3,00
2,00	2,00	2,00	3,00	2,00	4,00	3,00	3,00	2,00
2,00	2,00	2,00	2,00	4,00	4,00	3,00	3,00	2,00
2,00	2,00	2,00	3,00	0,00	3,00	3,00	3,00	3,00

2,00	2,00	2,00	2,00	4,00	4,00	3,00	2,00	1,00
2,00	2,00	2,00	2,00	4,00	4,00	4,00	4,00	3,00
2,00	2,00	2,00	2,00	0,00	4,00	1,00	2,00	1,00
2,00	2,00	2,00	2,00	4,00	3,00	2,00	3,00	1,00
2,00	2,00	2,00	2,00	4,00	3,00	2,00	4,00	1,00
2,00	2,00	2,00	3,00	4,00	3,00	2,00	3,00	1,00
2,00	2,00	2,00	2,00	0,00	4,00	3,00	4,00	2,00
2,00	2,00	2,00	3,00	3,00	4,00	3,00	4,00	4,00
2,00	2,00	2,00	1,00	6,00	4,00	4,00	4,00	4,00
2,00	2,00	2,00	1,00	6,00	3,00	3,00	2,00	4,00
2,00	1,00	2,00	1,00	5,00	4,00	4,00	4,00	4,00
2,00	2,00	2,00	1,00	5,00	3,00	3,00	3,00	1,00
2,00	2,00	2,00	1,00	6,00	4,00	3,00	4,00	2,00
2,00	2,00	2,00	1,00	6,00	3,00	3,00	3,00	1,00
2,00	2,00	2,00	1,00	8,00	3,00	2,00	3,00	2,00
2,00	2,00	1,00	1,00	8,00	2,00	2,00	2,00	2,00
2,00	2,00	2,00	1,00	8,00	3,00	3,00	3,00	3,00
2,00	2,00	2,00	1,00	7,00	4,00	3,00	3,00	1,00
2,00	2,00	2,00	1,00	8,00	2,00	3,00	3,00	2,00
2,00	2,00	2,00	1,00	8,00	3,00	3,00	3,00	1,00
2,00	2,00	2,00	1,00	8,00	3,00	3,00	3,00	1,00
2,00	2,00	2,00	1,00	7,00	3,00	2,00	2,00	1,00
2,00	2,00	2,00	1,00	7,00	3,00	1,00	2,00	2,00
2,00	2,00	2,00	1,00	7,00	3,00	2,00	4,00	2,00
2,00	4,00	2,00	1,00	7,00	2,00	2,00	1,00	1,00
1,00	2,00	2,00	1,00	2,00	2,00	1,00	2,00	2,00
1,00	2,00	2,00	1,00	2,00	2,00	2,00	4,00	4,00
1,00	2,00	2,00	4,00	2,00	1,00	2,00	2,00	3,00
1,00	3,00	2,00	4,00	2,00	4,00	1,00	1,00	1,00
1,00	2,00	2,00	4,00	2,00	4,00	2,00	2,00	2,00
1,00	2,00	2,00	4,00	2,00	2,00	1,00	2,00	3,00
1,00	2,00	2,00	2,00	8,00	4,00	3,00	4,00	3,00
1,00	2,00	1,00	4,00	2,00	2,00	1,00	2,00	2,00
1,00	3,00	1,00	4,00	2,00	3,00	2,00	3,00	2,00
2,00	3,00	2,00	4,00	1,00	4,00	2,00	2,00	3,00

Balls	Smoothie	Chocolate	Snack	NoWillingR	Neophobia	Environme	Sustainabl	Healthy
2,00	3,00	2,00	2,00	1,30	2,00	3,00	4,00	3,00
1,00	1,00	1,00	1,00	3,00	2,00	3,00	3,00	3,00
1,00	2,00	1,00	1,00	3,00	2,00	4,00	3,00	3,00
3,00	1,00	4,00	1,00	1,00	2,00	3,00	2,00	3,00
1,00	2,00	1,00	1,00	3,00	2,00	4,00	3,00	3,00
1,00	4,00	4,00	4,00	3,00	1,00	1,00	2,00	4,00
2,00	2,00	2,00	3,00	3,00	2,00	3,00	3,00	3,00
4,00	4,00	4,00	4,00	5,00	1,00	4,00	4,00	4,00
1,00	3,00	4,00	1,00	1,00	2,00	3,00	3,00	3,00
2,00	3,00	1,00	2,00	3,00	2,00	2,00	3,00	3,00
0,00	3,00	1,00	1,00	3,00	2,00	2,00	2,00	3,00
3,00	1,00	1,00	1,00	3,00	1,00	4,00	4,00	2,00
3,00	3,00	2,00	2,00	3,00	1,00	3,00	3,00	3,00
3,00	3,00	1,00	4,00	5,00	2,00	3,00	4,00	4,00
1,00	1,00	4,00	3,00	1,00	2,00	4,00	3,00	3,00
1,00	1,00	1,00	1,00	3,00	1,00	4,00	3,00	3,00
3,00	3,00	2,00	3,00	1,00	1,00	3,00	3,00	3,00
1,00	2,00	1,00	1,00	3,00	1,00	3,00	3,00	3,00
2,00	1,00	1,00	1,00	5,00	2,00	3,00	3,00	3,00
3,00	4,00	1,00	3,00	5,00	2,00	2,00	2,00	4,00
2,00	2,00	2,00	2,00	3,00	2,00	2,00	2,00	3,00
1,00	2,00	4,00	2,00	3,00	3,00	2,00	2,00	3,00
1,00	1,00	2,00	2,00	3,00	2,00	1,00	3,00	3,00
3,00	2,00	1,00	1,00	3,00	2,00	2,00	1,00	1,00
1,00	2,00	2,00	2,00	3,00	2,00	3,00	3,00	2,00
3,00	4,00	2,00	1,00	3,00	1,00	3,00	3,00	4,00
1,00	3,00	1,00	1,00	3,00	2,00	3,00	3,00	3,00
1,00	3,00	3,00	1,00	3,00	3,00	3,00	2,00	2,00
2,00	2,00	2,00	2,00	5,00	2,00	2,00	2,00	3,00
2,00	3,00	3,00	4,00	5,00	1,00	3,00	3,00	4,00
2,00	3,00	1,00	1,00	3,00	2,00	3,00	3,00	2,00
3,00	2,00	2,00	1,00	1,00	2,00	4,00	3,00	4,00
1,00	2,00	1,00	2,00	3,00	2,00	3,00	3,00	3,00
1,00	2,00	1,00	1,00	1,00	2,00	2,00	2,00	2,00
1,00	4,00	4,00	4,00	5,00	1,00	4,00	4,00	4,00
3,00	4,00	3,00	3,00	1,00	1,00	3,00	3,00	2,00
2,00	3,00	2,00	2,00	3,00	2,00	3,00	3,00	4,00
2,00	3,00	3,00	4,00	3,00	1,00	3,00	3,00	3,00
1,00	3,00	1,00	1,00	3,00	2,00	3,00	3,00	2,00
1,00	2,00	2,00	2,00	3,00	2,00	3,00	3,00	3,00
3,00	3,00	1,00	2,00	1,00	1,00	2,00	2,00	2,00
2,00	2,00	2,00	2,00	3,00	2,00	1,00	1,00	1,00
1,00	3,00	4,00	2,00	3,00	2,00	3,00	3,00	3,00
2,00	3,00	4,00	2,00	3,00	1,00	4,00	3,00	4,00
2,00	3,00	3,00	4,00	5,00	2,00	3,00	3,00	3,00
1,00	1,00	1,00	1,00	4,00	2,00	3,00	3,00	3,00

1,00	3,00	3,00	3,00	5,00	1,00	3,00	3,00	3,00
1,00	1,00	3,00	4,00	1,00	3,00	4,00	3,00	3,00
1,00	3,00	4,00	2,00	1,00	1,00	3,00	3,00	4,00
1,00	3,00	3,00	3,00	1,00	1,00	3,00	3,00	4,00
1,00	1,00	1,00	1,00	3,00	2,00	3,00	3,00	4,00
1,00	3,00	1,00	1,00	3,00	1,00	3,00	3,00	4,00
1,00	3,00	4,00	3,00	3,00	1,00	3,00	3,00	3,00
2,00	3,00	3,00	4,00	1,00	2,00	2,00	3,00	2,00
1,00	1,00	1,00	1,00	3,00	1,00	2,00	3,00	1,00
1,00	2,00	1,00	3,00	5,00	1,00	3,00	3,00	4,00
3,00	3,00	3,00	3,00	1,00	1,00	3,00	3,00	3,00
1,00	3,00	1,00	1,00	3,00	1,00	2,00	3,00	4,00
2,00	2,00	2,00	2,00	3,00	3,00	3,00	3,00	2,00
2,00	4,00	4,00	4,00	3,00	2,00	3,00	3,00	1,00
3,00	2,00	1,00	2,00	5,00	1,00	4,00	4,00	3,00
1,00	4,00	2,00	3,00	1,00	1,00	4,00	4,00	4,00
1,00	4,00	2,00	3,00	3,00	1,00	4,00	4,00	4,00
3,00	3,00	3,00	3,00	5,00	1,00	3,00	3,00	4,00
3,00	3,00	3,00	4,00	5,00	1,00	4,00	4,00	4,00
1,00	2,00	2,00	3,00	1,00	2,00	2,00	2,00	3,00
2,00	3,00	1,00	1,00	3,00	1,00	4,00	4,00	0,00
1,00	1,00	1,00	2,00	1,00	2,00	3,00	3,00	3,00
3,00	3,00	3,00	3,00	5,00	2,00	3,00	3,00	3,00
3,00	3,00	3,00	3,00	5,00	1,00	3,00	3,00	3,00
3,00	1,00	1,00	1,00	3,00	1,00	3,00	3,00	3,00
3,00	4,00	1,00	1,00	3,00	2,00	3,00	3,00	3,00
1,00	1,00	1,00	1,00	3,00	3,00	3,00	3,00	3,00
3,00	4,00	2,00	2,00	3,00	2,00	3,00	3,00	3,00
1,00	3,00	3,00	1,00	3,00	1,00	4,00	4,00	4,00
1,00	1,00	1,00	1,00	3,00	2,00	3,00	3,00	3,00
4,00	4,00	2,00	3,00	5,00	1,00	4,00	3,00	4,00
1,00	4,00	4,00	2,00	4,00	1,00	3,00	3,00	4,00
1,00	1,00	1,00	1,00	6,00	1,00	4,00	4,00	3,00
2,00	2,00	1,00	1,00	3,00	3,00	4,00	3,00	3,00
1,00	1,00	1,00	1,00	3,00	2,00	3,00	3,00	3,00
2,00	2,00	2,00	2,00	3,00	2,00	3,00	3,00	3,00
1,00	2,00	1,00	1,00	3,00	2,00	3,00	3,00	3,00
1,00	2,00	1,00	1,00	1,00	1,00	4,00	4,00	4,00
4,00	4,00	3,00	3,00	5,00	1,00	4,00	4,00	3,00
1,00	4,00	1,00	1,00	3,00	2,00	3,00	3,00	3,00
3,00	3,00	1,00	1,00	1,00	1,00	4,00	4,00	4,00
3,00	4,00	3,00	3,00	1,00	2,00	3,00	3,00	3,00
2,00	3,00	3,00	2,00	1,00	1,00	3,00	3,00	3,00
2,00	3,00	3,00	1,00	1,00	2,00	3,00	3,00	3,00
3,00	3,00	2,00	2,00	3,00	2,00	4,00	3,00	3,00
1,00	2,00	1,00	1,00	3,00	2,00	4,00	4,00	3,00
1,00	1,00	1,00	1,00	3,00	1,00	4,00	4,00	4,00

1,00	2,00	1,00	3,00	3,00	2,00	4,00	3,00	4,00
1,00	2,00	1,00	3,00	3,00	2,00	3,00	3,00	4,00
2,00	3,00	2,00	2,00	1,00	2,00	2,00	3,00	3,00
2,00	2,00	3,00	2,00	5,00	2,00	2,00	3,00	3,00
3,00	4,00	3,00	3,00	5,00	1,00	2,00	2,00	3,00
4,00	4,00	1,00	1,00	5,00	1,00	3,00	2,00	4,00
0,00	4,00	0,00	3,00	3,00	2,00	1,00	2,00	3,00
4,00	4,00	4,00	4,00	5,00	1,00	3,00	2,00	3,00
4,00	2,00	1,00	2,00	1,00	1,00	4,00	4,00	4,00
4,00	3,00	4,00	3,00	4,00	1,00	3,00	3,00	3,00
1,00	2,00	3,00	3,00	0,00	0,00	0,00	0,00	0,00
2,00	1,00	1,00	1,00	3,00	2,00	3,00	3,00	4,00
2,00	3,00	1,00	3,00	0,00	1,00	2,00	3,00	3,00
3,00	3,00	2,00	3,00	3,00	1,00	4,00	4,00	4,00
2,00	2,00	2,00	2,00	3,00	1,00	3,00	3,00	4,00
1,00	2,00	1,00	3,00	3,00	2,00	0,00	0,00	0,00
2,00	2,00	3,00	4,00	3,00	1,00	2,00	3,00	3,00
1,00	2,00	1,00	2,00	3,00	2,00	3,00	3,00	3,00
2,00	3,00	3,00	2,00	1,00	2,00	3,00	3,00	2,00
2,00	3,00	3,00	2,00	3,00	1,00	4,00	4,00	4,00
1,00	3,00	2,00	1,00	6,00	1,00	3,00	2,00	3,00
2,00	1,00	1,00	1,00	3,00	2,00	2,00	3,00	2,00
1,00	2,00	2,00	1,00	1,00	1,00	3,00	4,00	4,00
3,00	4,00	2,00	2,00	5,00	1,00	3,00	3,00	3,00
2,00	4,00	3,00	1,00	5,00	2,00	3,00	3,00	3,00
3,00	2,00	1,00	1,00	3,00	3,00	3,00	3,00	4,00
2,00	1,00	1,00	1,00	3,00	2,00	3,00	3,00	3,00
1,00	2,00	2,00	1,00	6,00	1,00	2,00	3,00	3,00
2,00	4,00	4,00	4,00	5,00	1,00	4,00	4,00	4,00
1,00	3,00	3,00	2,00	3,00	3,00	3,00	3,00	2,00
1,00	1,00	1,00	1,00	3,00	3,00	2,00	2,00	3,00
2,00	3,00	3,00	2,00	3,00	3,00	3,00	3,00	3,00
1,00	3,00	1,00	1,00	3,00	2,00	3,00	3,00	3,00
2,00	3,00	2,00	3,00	5,00	2,00	3,00	4,00	3,00
1,00	1,00	1,00	1,00	3,00	2,00	2,00	1,00	2,00
1,00	1,00	1,00	1,00	3,00	1,00	4,00	3,00	4,00
1,00	2,00	3,00	4,00	6,00	1,00	4,00	4,00	2,00
1,00	1,00	1,00	1,00	6,00	1,00	4,00	4,00	4,00
2,00	2,00	2,00	3,00	6,00	2,00	4,00	4,00	3,00
3,00	4,00	4,00	2,00	3,00	2,00	3,00	3,00	2,00
4,00	4,00	2,00	3,00	5,00	1,00	4,00	3,00	3,00
1,00	1,00	1,00	1,00	6,00	1,00	4,00	4,00	3,00
2,00	3,00	2,00	1,00	5,00	2,00	3,00	2,00	3,00
2,00	2,00	2,00	3,00	1,00	1,00	3,00	3,00	3,00
3,00	4,00	3,00	2,00	5,00	1,00	4,00	4,00	4,00
4,00	4,00	4,00	4,00	4,00	2,00	4,00	4,00	3,00
2,00	3,00	1,00	2,00	5,00	1,00	3,00	3,00	3,00

2,00	3,00	3,00	3,00	4,00	2,00	3,00	3,00	3,00
2,00	3,00	2,00	4,00	1,00	2,00	4,00	4,00	4,00
1,00	2,00	1,00	1,00	3,00	2,00	3,00	3,00	3,00
2,00	4,00	1,00	4,00	5,00	1,00	3,00	3,00	3,00
1,00	1,00	2,00	3,00	3,00	2,00	3,00	3,00	3,00
1,00	2,00	3,00	1,00	3,00	2,00	3,00	3,00	4,00
1,00	2,00	3,00	1,00	3,00	2,00	3,00	3,00	4,00
3,00	2,00	2,00	1,00	6,00	2,00	3,00	3,00	2,00
1,00	1,00	1,00	1,00	3,00	2,00	4,00	3,00	3,00
1,00	1,00	2,00	1,00	3,00	2,00	3,00	3,00	3,00
1,00	1,00	2,00	1,00	3,00	3,00	2,00	2,00	2,00
2,00	3,00	3,00	2,00	3,00	1,00	1,00	2,00	3,00
1,00	3,00	1,00	1,00	5,00	1,00	3,00	2,00	3,00
2,00	2,00	2,00	2,00	1,00	3,00	3,00	2,00	2,00
1,00	3,00	1,00	1,00	5,00	2,00	2,00	2,00	2,00
2,00	2,00	1,00	2,00	3,00	3,00	2,00	2,00	2,00
2,00	2,00	1,00	1,00	3,00	2,00	3,00	3,00	3,00
2,00	2,00	2,00	2,00	1,00	1,00	2,00	3,00	3,00
1,00	1,00	1,00	1,00	2,00	2,00	3,00	3,00	3,00
2,00	3,00	4,00	1,00	3,00	2,00	3,00	3,00	3,00
4,00	4,00	4,00	4,00	5,00	1,00	4,00	3,00	4,00
1,00	3,00	3,00	4,00	1,00	1,00	4,00	3,00	4,00
4,00	4,00	4,00	4,00	5,00	1,00	1,00	2,00	3,00
4,00	4,00	4,00	4,00	5,00	1,00	3,00	2,00	2,00
2,00	3,00	3,00	3,00	5,00	1,00	4,00	3,00	3,00
2,00	2,00	1,00	1,00	3,00	2,00	3,00	4,00	3,00
2,00	2,00	2,00	1,00	6,00	1,00	4,00	4,00	4,00
1,00	2,00	2,00	1,00	3,00	2,00	3,00	3,00	3,00
1,00	2,00	2,00	1,00	1,00	1,00	4,00	4,00	3,00
2,00	2,00	2,00	2,00	3,00	3,00	3,00	3,00	2,00
2,00	1,00	2,00	2,00	3,00	2,00	2,00	2,00	4,00
1,00	1,00	1,00	1,00	3,00	2,00	3,00	3,00	4,00
2,00	2,00	1,00	1,00	3,00	2,00	3,00	2,00	2,00
2,00	3,00	1,00	1,00	3,00	2,00	3,00	3,00	2,00
1,00	1,00	1,00	1,00	3,00	3,00	1,00	1,00	1,00
3,00	3,00	1,00	2,00	3,00	2,00	3,00	2,00	1,00
1,00	3,00	3,00	3,00	3,00	3,00	3,00	3,00	3,00
2,00	2,00	2,00	2,00	3,00	2,00	3,00	3,00	3,00
2,00	3,00	3,00	1,00	1,00	1,00	3,00	3,00	3,00
1,00	4,00	4,00	4,00	2,00	3,00	3,00	3,00	2,00
2,00	2,00	2,00	2,00	1,00	2,00	3,00	0,00	3,00
2,00	3,00	1,00	3,00	5,00	1,00	4,00	3,00	4,00
2,00	3,00	2,00	2,00	1,00	1,00	2,00	3,00	3,00
2,00	2,00	3,00	3,00	1,00	2,00	3,00	3,00	4,00
1,00	3,00	4,00	3,00	1,00	2,00	3,00	2,00	4,00
2,00	1,00	2,00	4,00	3,00	3,00	1,00	1,00	1,00
2,00	2,00	2,00	3,00	1,00	2,00	2,00	2,00	2,00

2,00	1,00	2,00	1,00	6,00	2,00	3,00	3,00	3,00
4,00	4,00	4,00	4,00	5,00	1,00	4,00	4,00	3,00
0,00	0,00	2,00	2,00	3,00	2,00	2,00	2,00	3,00
3,00	2,00	3,00	3,00	6,00	2,00	3,00	3,00	3,00
2,00	3,00	3,00	3,00	3,00	1,00	3,00	3,00	2,00
2,00	2,00	1,00	1,00	3,00	1,00	2,00	2,00	3,00
2,00	3,00	3,00	3,00	1,00	3,00	4,00	4,00	4,00
3,00	4,00	4,00	2,00	3,00	1,00	3,00	2,00	4,00
2,00	3,00	2,00	3,00	4,00	1,00	3,00	3,00	2,00
2,00	2,00	3,00	3,00	1,00	1,00	3,00	2,00	3,00
3,00	4,00	2,00	2,00	3,00	1,00	4,00	4,00	3,00
1,00	2,00	1,00	2,00	3,00	2,00	3,00	3,00	1,00
1,00	3,00	1,00	1,00	1,00	2,00	3,00	3,00	3,00
1,00	3,00	1,00	1,00	1,00	2,00	3,00	3,00	3,00
1,00	2,00	2,00	1,00	3,00	2,00	3,00	3,00	3,00
2,00	2,00	2,00	2,00	1,00	3,00	3,00	3,00	3,00
2,00	1,00	2,00	2,00	1,00	2,00	3,00	2,00	2,00
2,00	3,00	2,00	2,00	3,00	2,00	3,00	3,00	2,00
3,00	1,00	3,00	1,00	3,00	3,00	3,00	3,00	3,00
2,00	3,00	3,00	3,00	1,00	1,00	3,00	2,00	4,00
2,00	2,00	2,00	2,00	1,00	1,00	2,00	2,00	3,00
1,00	1,00	1,00	2,00	3,00	2,00	3,00	3,00	2,00
2,00	2,00	2,00	2,00	3,00	1,00	3,00	2,00	4,00
1,00	2,00	1,00	2,00	3,00	2,00	3,00	3,00	0,00
1,00	1,00	1,00	1,00	2,00	3,00	2,00	3,00	3,00
1,00	3,00	2,00	2,00	1,00	3,00	3,00	3,00	2,00
1,00	3,00	3,00	3,00	2,00	2,00	4,00	3,00	3,00
2,00	3,00	3,00	2,00	1,00	2,00	2,00	3,00	3,00
1,00	3,00	1,00	1,00	3,00	2,00	2,00	2,00	3,00
1,00	3,00	2,00	2,00	1,00	2,00	3,00	3,00	3,00
2,00	3,00	2,00	2,00	1,00	2,00	2,00	2,00	3,00
2,00	4,00	2,00	3,00	3,00	1,00	4,00	4,00	4,00
2,00	2,00	2,00	2,00	3,00	1,00	3,00	3,00	3,00
1,00	1,00	2,00	1,00	3,00	2,00	4,00	4,00	4,00
1,00	1,00	2,00	4,00	6,00	1,00	4,00	4,00	4,00

AnimalsBer	Enviromen	MeatProdu	MydietBen	Diet	NoWillingR	Familiar	New	Whole
3,00	3,00	2,00	0,00	1,00	1,00	2,00	2,50	2,00
2,00	2,00	2,00	1,00	1,00	2,00	1,00	1,00	1,00
4,00	3,00	2,00	1,00	1,00	3,00	2,50	1,50	1,00
3,00	4,00	3,00	3,00	2,00	4,00	2,50	2,00	2,50
4,00	4,00	3,00	2,00	1,00	5,00	2,00	1,50	1,00
1,00	1,00	1,00	1,00	1,00	6,00	4,00	2,50	4,00
3,00	3,00	3,00	2,00	1,00	7,00	3,00	2,00	2,50
4,00	4,00	4,00	4,00	1,00	8,00	4,00	4,00	4,00
3,00	4,00	3,00	3,00	1,00	9,00	2,50	2,00	2,50
3,00	3,00	3,00	2,00	1,00	10,00	3,00	2,50	1,50
1,00	2,00	2,00	3,00	1,00	11,00	2,00	1,50	1,00
4,00	4,00	2,00	2,00	1,00	12,00	1,50	2,00	1,00
4,00	4,00	3,00	3,00	1,00	13,00	3,00	3,00	2,00
4,00	4,00	4,00	4,00	1,00	14,00	2,00	3,00	2,50
3,00	3,00	4,00	4,00	1,00	15,00	3,00	1,00	3,50
4,00	4,00	4,00	1,00	1,00	16,00	2,00	1,00	1,00
4,00	4,00	3,00	4,00	1,00	17,00	2,50	3,00	2,50
3,00	3,00	1,00	2,00	1,00	18,00	1,50	1,50	1,00
3,00	3,00	2,00	2,00	1,00	19,00	2,50	1,50	1,00
2,00	3,00	2,00	4,00	1,00	20,00	3,00	3,50	2,00
3,00	3,00	2,00	1,00	1,00	21,00	3,00	2,00	2,00
3,00	3,00	3,00	1,00	1,00	22,00	3,50	1,50	3,00
3,00	2,00	1,00	3,00	1,00	23,00	3,00	1,00	2,00
4,00	4,00	4,00	2,00	1,00	24,00	4,00	2,50	1,00
2,00	3,00	3,00	2,00	1,00	25,00	3,00	1,50	2,00
4,00	4,00	3,00	4,00	1,00	26,00	4,00	3,50	1,50
3,00	3,00	2,00	2,00	1,00	27,00	2,50	2,00	1,00
3,00	3,00	3,00	3,00	1,00	28,00	2,50	2,00	2,00
4,00	4,00	4,00	2,00	1,00	29,00	3,00	2,00	2,00
4,00	4,00	3,00	3,00	1,00	30,00	2,50	2,50	3,50
3,00	3,00	3,00	2,00	1,00	31,00	2,50	2,50	1,00
4,00	3,00	3,00	3,00	1,00	32,00	3,50	2,50	1,50
2,00	4,00	1,00	2,00	1,00	33,00	2,00	1,50	1,50
4,00	3,00	2,00	3,00	1,00	34,00	1,50	1,50	1,00
4,00	4,00	4,00	0,00	0,00	35,00	2,00	2,50	4,00
4,00	4,00	4,00	0,00	0,00	36,00	3,50	3,50	3,00
3,00	3,00	2,00	3,00	1,00	37,00	2,50	2,50	2,00
4,00	4,00	4,00	3,00	1,00	38,00	2,00	2,50	3,50
3,00	3,00	2,00	2,00	1,00	39,00	2,00	2,00	1,00
3,00	3,00	1,00	1,00	1,00	40,00	2,00	1,50	2,00
4,00	3,00	2,00	3,00	1,00	41,00	1,50	3,00	1,50
3,00	3,00	3,00	1,00	1,00	42,00	2,00	2,00	2,00
3,00	2,00	3,00	1,00	1,00	43,00	3,00	2,00	3,00
3,00	3,00	3,00	3,00	1,00	44,00	4,00	2,50	3,00
3,00	3,00	3,00	3,00	1,00	45,00	4,00	2,50	3,50
4,00	4,00	2,00	2,00	1,00	46,00	1,00	1,00	1,00

3,00	3,00	3,00	3,00	2,00	47,00	3,00	2,00	3,00
4,00	4,00	3,00	3,00	1,00	48,00	2,50	1,00	3,50
4,00	3,00	3,00	4,00	1,00	49,00	2,50	2,00	3,00
3,00	3,00	3,00	3,00	1,00	50,00	3,00	2,00	3,00
3,00	3,00	2,00	1,00	1,00	51,00	1,00	1,00	1,00
4,00	3,00	1,00	1,00	1,00	52,00	2,00	2,00	1,00
4,00	4,00	2,00	0,00	1,00	53,00	3,50	2,00	3,50
2,00	3,00	2,00	3,00	1,00	54,00	4,00	2,50	3,50
3,00	2,00	2,00	4,00	1,00	55,00	1,00	1,00	1,00
4,00	3,00	3,00	4,00	1,00	56,00	1,00	1,50	2,00
3,00	3,00	3,00	3,00	2,00	57,00	3,00	3,00	3,00
3,00	2,00	2,00	3,00	2,00	58,00	2,00	2,00	1,00
3,00	3,00	3,00	2,00	1,00	59,00	1,50	2,00	2,00
2,00	3,00	3,00	1,00	0,00	60,00	4,00	3,00	4,00
4,00	4,00	4,00	4,00	1,00	61,00	3,00	2,50	1,50
3,00	3,00	2,00	3,00	3,00	62,00	3,00	2,50	2,50
1,00	1,00	1,00	1,00	3,00	63,00	1,50	2,50	2,50
4,00	4,00	4,00	4,00	1,00	64,00	3,00	3,00	3,00
3,00	3,00	2,00	4,00	1,00	65,00	3,50	3,00	3,50
3,00	2,00	3,00	3,00	1,00	66,00	3,00	1,50	2,50
4,00	4,00	4,00	4,00	3,00	67,00	2,00	2,50	1,00
3,00	3,00	2,00	2,00	1,00	68,00	1,50	1,00	1,50
3,00	3,00	3,00	3,00	1,00	69,00	2,50	3,00	3,00
3,00	3,00	3,00	3,00	1,00	70,00	3,00	3,00	3,00
3,00	3,00	2,00	2,00	2,00	71,00	1,00	2,00	1,00
4,00	4,00	3,00	2,00	2,00	72,00	2,00	3,50	1,00
2,00	2,00	1,00	1,00	1,00	73,00	1,00	1,00	1,00
4,00	4,00	3,00	3,00	1,00	74,00	3,00	3,50	2,00
2,00	2,00	3,00	3,00	3,00	75,00	2,00	2,00	2,00
2,00	2,00	2,00	2,00	1,00	76,00	1,00	1,00	1,00
4,00	3,00	3,00	2,00	1,00	77,00	4,00	4,00	2,50
1,00	1,00	2,00	3,00	1,00	78,00	2,50	2,50	3,00
1,00	1,00	1,00	1,00	3,00	79,00	1,00	1,00	1,00
2,00	3,00	2,00	3,00	1,00	80,00	2,50	2,00	1,00
3,00	3,00	3,00	1,00	1,00	81,00	2,00	1,00	1,00
3,00	2,00	2,00	3,00	2,00	82,00	2,00	2,00	2,00
3,00	3,00	2,00	1,00	1,00	83,00	2,00	1,50	1,00
4,00	3,00	4,00	3,00	1,00	84,00	1,50	1,50	1,00
3,00	4,00	3,00	3,00	2,00	85,00	3,50	4,00	3,00
4,00	4,00	3,00	2,00	1,00	86,00	2,50	2,50	1,00
4,00	4,00	4,00	3,00	1,00	87,00	2,00	3,00	1,00
3,00	3,00	3,00	3,00	1,00	88,00	3,50	3,50	3,00
3,00	3,00	3,00	2,00	1,00	89,00	4,00	2,50	2,50
3,00	3,00	2,00	1,00	1,00	90,00	2,50	2,50	2,00
4,00	4,00	4,00	4,00	2,00	91,00	3,50	3,00	2,00
4,00	4,00	2,00	2,00	3,00	92,00	2,00	1,50	1,00
4,00	4,00	1,00	1,00	3,00	93,00	1,00	1,00	1,00

3,00	4,00	2,00	3,00	1,00	94,00	2,50	1,50	2,00
3,00	3,00	2,00	3,00	1,00	95,00	2,50	1,50	2,00
3,00	3,00	3,00	3,00	1,00	96,00	2,50	2,50	2,00
2,00	3,00	4,00	3,00	1,00	97,00	2,00	2,00	2,50
2,00	2,00	3,00	3,00	1,00	98,00	3,00	3,50	3,00
3,00	1,00	1,00	3,00	2,00	99,00	4,00	4,00	1,00
4,00	3,00	2,00	1,00	1,00	100,00	2,50	2,00	1,50
4,00	3,00	3,00	4,00	1,00	101,00	4,00	4,00	4,00
4,00	4,00	3,00	4,00	1,00	102,00	3,00	3,00	1,50
3,00	3,00	4,00	4,00	1,00	103,00	4,00	3,50	3,50
0,00	0,00	0,00	3,00	1,00	104,00	3,50	1,50	3,00
4,00	3,00	3,00	3,00	2,00	105,00	3,00	1,50	1,00
3,00	3,00	3,00	3,00	0,00	106,00	3,50	2,50	2,00
4,00	4,00	3,00	3,00	1,00	107,00	2,50	3,00	2,50
3,00	3,00	3,00	3,00	1,00	108,00	2,00	2,00	2,00
3,00	3,00	3,00	3,00	1,00	109,00	2,50	1,50	2,00
3,00	3,00	3,00	2,00	1,00	110,00	2,50	2,00	3,50
3,00	3,00	3,00	2,00	1,00	111,00	2,00	1,50	1,50
3,00	3,00	3,00	3,00	1,00	112,00	3,00	2,50	2,50
3,00	3,00	2,00	3,00	2,00	113,00	2,50	2,50	2,50
1,00	1,00	1,00	1,00	3,00	114,00	3,50	2,00	1,50
3,00	3,00	3,00	2,00	1,00	115,00	2,50	1,50	1,00
3,00	3,00	3,00	3,00	3,00	116,00	2,00	1,50	1,50
3,00	3,00	3,00	3,00	1,00	117,00	3,50	3,50	2,00
4,00	4,00	3,00	2,00	1,00	118,00	3,50	3,00	2,00
3,00	2,00	3,00	3,00	1,00	119,00	2,50	2,50	1,00
3,00	3,00	2,00	1,00	1,00	120,00	2,00	1,50	1,00
3,00	4,00	3,00	3,00	1,00	121,00	2,00	1,50	1,50
1,00	2,00	3,00	4,00	2,00	122,00	3,50	3,00	4,00
3,00	3,00	2,00	2,00	1,00	123,00	1,00	2,00	2,50
3,00	3,00	2,00	2,00	1,00	124,00	1,00	1,00	1,00
3,00	3,00	3,00	2,00	1,00	125,00	2,50	2,50	2,50
4,00	4,00	1,00	1,00	1,00	126,00	2,00	2,00	1,00
3,00	4,00	4,00	3,00	2,00	127,00	2,50	2,50	2,50
1,00	1,00	1,00	1,00	1,00	128,00	1,50	1,00	1,00
4,00	4,00	1,00	2,00	1,00	129,00	1,50	1,00	1,00
4,00	4,00	3,00	4,00	2,00	130,00	2,50	1,50	3,50
1,00	2,00	2,00	2,00	4,00	131,00	1,00	1,00	1,00
1,00	1,00	1,00	2,00	3,00	132,00	2,00	2,00	2,50
3,00	4,00	4,00	3,00	2,00	133,00	2,50	3,50	3,00
4,00	4,00	4,00	3,00	2,00	134,00	4,00	4,00	2,50
3,00	3,00	3,00	3,00	3,00	135,00	1,00	1,00	1,00
3,00	3,00	3,00	3,00	1,00	136,00	3,00	2,50	1,50
3,00	3,00	3,00	3,00	3,00	137,00	2,50	2,00	2,50
4,00	4,00	4,00	4,00	1,00	138,00	2,50	3,50	2,50
3,00	3,00	3,00	2,00	1,00	139,00	4,00	4,00	4,00
3,00	3,00	3,00	2,00	2,00	140,00	2,50	2,50	1,50

2,00	3,00	3,00	3,00	1,00	141,00	2,50	2,50	3,00
3,00	4,00	4,00	4,00	2,00	142,00	1,00	2,50	3,00
2,00	3,00	2,00	2,00	3,00	143,00	1,50	1,50	1,00
3,00	3,00	3,00	3,00	1,00	144,00	3,50	3,00	2,50
3,00	3,00	3,00	2,00	2,00	145,00	2,50	1,00	2,50
3,00	4,00	4,00	4,00	1,00	146,00	2,50	1,50	2,00
2,00	2,00	2,00	3,00	1,00	147,00	2,00	1,50	2,00
3,00	3,00	3,00	3,00	1,00	148,00	4,00	2,50	1,50
2,00	2,00	2,00	2,00	1,00	149,00	1,00	1,00	1,00
3,00	3,00	2,00	2,00	1,00	150,00	2,00	1,00	1,50
3,00	3,00	2,00	2,00	1,00	151,00	2,50	1,00	1,50
3,00	3,00	3,00	3,00	1,00	152,00	3,00	2,50	2,50
3,00	3,00	3,00	3,00	1,00	153,00	3,50	2,00	1,00
3,00	3,00	3,00	0,00	0,00	154,00	2,50	2,00	2,00
3,00	3,00	3,00	2,00	1,00	155,00	2,50	2,00	1,00
2,00	3,00	3,00	2,00	1,00	156,00	3,50	2,00	1,50
3,00	4,00	2,00	3,00	1,00	157,00	3,00	2,00	1,00
3,00	3,00	2,00	3,00	1,00	158,00	3,00	2,00	2,00
2,00	2,00	2,00	2,00	1,00	159,00	1,00	1,00	1,00
4,00	4,00	4,00	4,00	1,00	160,00	3,50	2,50	2,50
4,00	4,00	3,00	3,00	1,00	161,00	4,00	4,00	4,00
4,00	3,00	4,00	4,00	2,00	162,00	3,00	2,00	3,50
4,00	3,00	2,00	4,00	1,00	163,00	4,00	4,00	4,00
4,00	4,00	4,00	4,00	1,00	164,00	4,00	4,00	4,00
4,00	4,00	4,00	4,00	1,00	165,00	3,00	2,50	3,00
3,00	3,00	3,00	1,00	1,00	166,00	2,00	2,00	1,00
1,00	1,00	1,00	2,00	4,00	167,00	1,50	2,00	1,50
3,00	3,00	2,00	1,00	1,00	168,00	1,50	1,50	1,50
3,00	3,00	3,00	2,00	1,00	169,00	3,00	1,50	1,50
3,00	3,00	3,00	3,00	2,00	170,00	1,50	2,00	2,00
3,00	2,00	2,00	4,00	1,00	171,00	2,50	1,50	2,00
2,00	2,00	2,00	1,00	1,00	172,00	1,50	1,00	1,00
4,00	4,00	2,00	1,00	1,00	173,00	2,50	2,00	1,00
4,00	4,00	2,00	2,00	1,00	174,00	2,50	2,50	1,00
4,00	4,00	1,00	1,00	1,00	175,00	1,00	1,00	1,00
4,00	3,00	3,00	2,00	1,00	176,00	2,00	3,00	1,50
3,00	2,00	3,00	2,00	1,00	177,00	3,50	2,00	3,00
3,00	2,00	3,00	3,00	1,00	178,00	2,00	2,00	2,00
0,00	3,00	3,00	3,00	1,00	179,00	2,00	2,50	2,00
3,00	2,00	3,00	3,00	2,00	180,00	4,00	2,50	4,00
4,00	4,00	2,00	3,00	1,00	181,00	2,50	2,00	2,00
4,00	4,00	3,00	0,00	0,00	182,00	2,00	2,50	2,00
4,00	4,00	3,00	3,00	1,00	183,00	3,00	2,50	2,00
3,00	3,00	0,00	3,00	1,00	184,00	3,00	2,00	3,00
4,00	4,00	3,00	4,00	1,00	185,00	2,50	2,00	3,50
4,00	2,00	2,00	3,00	0,00	186,00	2,50	1,50	3,00
3,00	3,00	3,00	3,00	1,00	187,00	3,00	2,00	2,50

3,00	4,00	3,00	2,00	1,00	188,00	1,50	1,50	1,50
4,00	4,00	4,00	4,00	1,00	189,00	3,50	4,00	4,00
3,00	3,00	2,00	2,00	1,00	190,00	1,50	0,00	2,00
3,00	0,00	3,00	2,00	1,00	191,00	2,00	2,50	3,00
3,00	3,00	3,00	4,00	1,00	192,00	2,50	2,50	3,00
3,00	1,00	1,00	2,00	1,00	193,00	2,00	2,00	1,00
3,00	4,00	4,00	3,00	1,00	194,00	3,00	2,50	3,00
4,00	4,00	3,00	3,00	1,00	195,00	4,00	3,50	3,00
4,00	4,00	4,00	3,00	1,00	196,00	4,00	2,50	2,50
4,00	4,00	3,00	3,00	1,00	197,00	3,00	2,00	3,00
4,00	4,00	4,00	3,00	3,00	198,00	4,00	3,50	2,00
4,00	4,00	2,00	3,00	1,00	199,00	2,00	1,50	1,50
4,00	4,00	2,00	2,00	1,00	200,00	3,00	2,00	1,00
4,00	4,00	2,00	1,00	1,00	201,00	2,00	2,00	1,00
3,00	4,00	3,00	0,00	0,00	202,00	2,50	1,50	1,50
3,00	3,00	2,00	2,00	1,00	203,00	2,00	2,00	2,00
3,00	3,00	2,00	3,00	1,00	204,00	3,00	1,50	2,00
3,00	3,00	3,00	2,00	1,00	205,00	2,00	2,50	2,00
3,00	3,00	3,00	3,00	2,00	206,00	2,50	2,00	2,00
3,00	3,00	2,00	4,00	1,00	207,00	2,00	2,50	3,00
3,00	2,00	2,00	3,00	1,00	208,00	2,00	2,00	2,00
3,00	3,00	3,00	2,00	1,00	209,00	1,50	1,00	1,50
3,00	3,00	2,00	1,00	1,00	210,00	2,00	2,00	2,00
3,00	3,00	3,00	2,00	1,00	211,00	3,00	1,50	1,50
2,00	2,00	2,00	2,00	1,00	212,00	1,00	1,00	1,00
2,00	2,00	2,00	1,00	1,00	213,00	2,00	2,00	2,00
3,00	4,00	4,00	4,00	2,00	214,00	4,00	2,00	3,00
3,00	4,00	2,00	2,00	1,00	215,00	2,50	2,50	2,50
4,00	4,00	1,00	1,00	1,00	216,00	1,00	2,00	1,00
4,00	3,00	3,00	3,00	1,00	217,00	2,00	2,00	2,00
2,00	3,00	3,00	2,00	1,00	218,00	2,50	2,50	2,00
4,00	4,00	4,00	3,00	1,00	219,00	3,50	3,00	2,50
3,00	3,00	3,00	2,00	1,00	220,00	2,00	2,00	2,00
4,00	4,00	4,00	3,00	1,00	221,00	2,50	1,00	1,50
4,00	4,00	3,00	3,00	2,00	222,00	2,50	1,00	3,00

InfoFood	InfoFeed	Motivation
1,00	3,00	5,00
1,50	2,00	4,50
1,50	3,50	5,00
3,00	3,50	4,00
2,50	4,00	5,00
1,00	1,00	3,50
2,50	3,00	4,50
4,00	4,00	6,00
3,00	3,50	4,50
2,50	3,00	4,00
2,50	1,50	3,50
2,00	4,00	5,00
3,00	4,00	4,50
4,00	4,00	5,50
4,00	3,00	5,00
2,50	4,00	5,00
3,50	4,00	4,50
1,50	3,00	4,50
2,00	3,00	4,50
3,00	2,50	4,00
1,50	3,00	3,50
2,00	3,00	3,50
2,00	2,50	3,50
3,00	4,00	2,00
2,50	2,50	4,00
3,50	4,00	5,00
2,00	3,00	4,50
3,00	3,00	3,50
3,00	4,00	3,50
3,00	4,00	5,00
2,50	3,00	4,00
3,00	3,50	5,50
1,50	3,00	4,50
2,50	3,50	3,00
2,00	4,00	6,00
2,00	4,00	4,00
2,50	3,00	5,00
3,50	4,00	4,50
2,00	3,00	4,00
1,00	3,00	4,50
2,50	3,50	3,00
2,00	3,00	1,50
2,00	2,50	4,50
3,00	3,00	5,50
3,00	3,00	4,50
2,00	4,00	4,50

3,00	3,00	4,50
3,00	4,00	5,00
3,50	3,50	5,00
3,00	3,00	5,00
1,50	3,00	5,00
1,00	3,50	5,00
1,00	4,00	4,50
2,50	2,50	3,50
3,00	2,50	3,00
3,50	3,50	5,00
3,00	3,00	4,50
2,50	2,50	4,50
2,50	3,00	4,00
2,00	2,50	3,50
4,00	4,00	5,50
2,50	3,00	6,00
1,00	1,00	6,00
4,00	4,00	5,00
3,00	3,00	6,00
3,00	2,50	3,50
4,00	4,00	4,00
2,00	3,00	4,50
3,00	3,00	4,50
3,00	3,00	4,50
2,00	3,00	4,50
2,50	4,00	4,50
1,00	2,00	4,50
3,00	4,00	4,50
3,00	2,00	6,00
2,00	2,00	4,50
2,50	3,50	5,50
2,50	1,00	5,00
1,00	1,00	5,50
2,50	2,50	5,00
2,00	3,00	4,50
2,50	2,50	4,50
1,50	3,00	4,50
3,50	3,50	6,00
3,00	3,50	5,50
2,50	4,00	4,50
3,50	4,00	6,00
3,00	3,00	4,50
2,50	3,00	4,50
1,50	3,00	4,50
4,00	4,00	5,00
2,00	4,00	5,50
1,00	4,00	6,00

2,50	3,50	5,50
2,50	3,00	5,00
3,00	3,00	4,00
3,50	2,50	4,00
3,00	2,00	3,50
2,00	2,00	4,50
1,50	3,50	3,00
3,50	3,50	4,00
3,50	4,00	6,00
4,00	3,00	4,50
1,50	0,00	0,00
3,00	3,50	5,00
3,00	3,00	4,00
3,00	4,00	6,00
3,00	3,00	5,00
3,00	3,00	0,00
2,50	3,00	4,00
2,50	3,00	4,50
3,00	3,00	4,00
2,50	3,00	6,00
1,00	1,00	4,00
2,50	3,00	3,50
3,00	3,00	5,50
3,00	3,00	4,50
2,50	4,00	4,50
3,00	2,50	5,00
1,50	3,00	4,50
3,00	3,50	4,00
3,50	1,50	6,00
2,00	3,00	4,00
2,00	3,00	3,50
2,50	3,00	4,50
1,00	4,00	4,50
3,50	3,50	5,00
1,00	1,00	2,50
1,50	4,00	5,50
3,50	4,00	5,00
2,00	1,50	6,00
1,50	1,00	5,50
3,50	3,50	4,00
3,50	4,00	5,00
3,00	3,00	5,50
3,00	3,00	4,00
3,00	3,00	4,50
4,00	4,00	6,00
2,50	3,00	5,50
2,50	3,00	4,50

3,00	2,50	4,50
4,00	3,50	6,00
2,00	2,50	4,50
3,00	3,00	4,50
2,50	3,00	4,50
4,00	3,50	5,00
2,50	2,00	5,00
3,00	3,00	4,00
2,00	2,00	5,00
2,00	3,00	4,50
2,00	3,00	3,00
3,00	3,00	3,00
3,00	3,00	4,00
1,50	3,00	3,50
2,50	3,00	3,00
2,50	2,50	3,00
2,50	3,50	4,50
2,50	3,00	4,00
2,00	2,00	4,50
4,00	4,00	4,50
3,00	4,00	5,50
4,00	3,50	5,50
3,00	3,50	3,00
4,00	4,00	3,50
4,00	4,00	5,00
2,00	3,00	5,00
1,50	1,00	6,00
1,50	3,00	4,50
2,50	3,00	5,50
3,00	3,00	4,00
3,00	2,50	4,00
1,50	2,00	5,00
1,50	4,00	3,50
2,00	4,00	4,00
1,00	4,00	1,50
2,50	3,50	3,00
2,50	2,50	4,50
3,00	2,50	4,50
3,00	1,50	4,50
3,00	2,50	4,00
2,50	4,00	3,00
1,50	4,00	5,50
3,00	4,00	4,00
1,50	3,00	5,00
3,50	4,00	4,50
2,50	3,00	1,50
3,00	3,00	3,00

2,50	3,50	4,50
4,00	4,00	5,50
2,00	3,00	3,50
2,50	1,50	4,50
3,50	3,00	4,00
1,50	2,00	3,50
3,50	3,50	6,00
3,00	4,00	4,50
3,50	4,00	4,00
3,00	4,00	4,00
3,50	4,00	5,50
2,50	4,00	3,50
2,00	4,00	4,50
1,50	4,00	4,50
1,50	3,50	4,50
2,00	3,00	4,50
2,50	3,00	3,50
2,50	3,00	4,00
3,00	3,00	4,50
3,00	3,00	4,50
2,50	2,50	3,50
2,50	3,00	4,00
1,50	3,00	4,50
2,50	3,00	3,00
2,00	2,00	4,00
1,50	2,00	4,00
4,00	3,50	5,00
2,00	3,50	4,00
1,00	4,00	3,50
3,00	3,50	4,50
2,50	2,50	3,50
3,50	4,00	6,00
2,50	3,00	4,50
3,50	4,00	6,00
3,00	4,00	6,00
